# A Daphnane Diterpenoid Isolated from *Wikstroemia polyantha* Induces an Inflammatory Response and Modulates miRNA Activity

**DOI:** 10.1371/journal.pone.0039621

**Published:** 2012-06-26

**Authors:** Anthony Khong, Roberto Forestieri, David E. Williams, Brian O. Patrick, Andrea Olmstead, Victoria Svinti, Emily Schaeffer, François Jean, Michel Roberge, Raymond J. Andersen, Eric Jan

**Affiliations:** 1 Department of Biochemistry and Molecular Biology, University of British Columbia, Vancouver, British Columbia, Canada; 2 Department of Chemistry and Department of Earth and Ocean Sciences, University of British Columbia, Vancouver, British Columbia, Canada; 3 Department of Microbiology and Immunology, University of British Columbia, Vancouver, British Columbia, Canada; National Institutes of Health, United States of America

## Abstract

MicroRNAs (miRNAs) are endogenously expressed single-stranded ∼21–23 nucleotide RNAs that inhibit gene expression post-transcriptionally by binding imperfectly to elements usually within the 3′untranslated region (3′UTR) of mRNAs. Small interfering RNAs (siRNAs) mediate site-specific cleavage by binding with perfect complementarity to RNA. Here, a cell-based miRNA reporter system was developed to screen for compounds from marine and plant extracts that inhibit miRNA or siRNA activity. The daphnane diterpenoid genkwanine M (GENK) isolated from the plant *Wikstroemia polyantha* induces an early inflammatory response and can moderately inhibit miR-122 activity in the liver Huh-7 cell line. GENK does not alter miR-122 levels nor does it directly inhibit siRNA activity in an *in vitro* cleavage assay. Finally, we demonstrate that GENK can inhibit HCV infection in Huh-7 cells. In summary, the development of the cell-based miRNA sensor system should prove useful in identifying compounds that affect miRNA/siRNA activity.

## Introduction

MicroRNAs (miRNAs) are small endogenous non-coding single-stranded RNAs (typically 21–23 nucleotide long) that act posttranscriptionally to repress translation [1]–[5] and/or induce mRNA decay [6]–[8]. miRNAs play significant roles in a number of cellular processes and their misregulation has been linked to many pathological states, including cancer [9], [10], diabetes [11], [12], neurodegenerative disease [13] and viral infections [14]–[16]. To date, over 1900 unique mature miRNA sequences have been identified in *Homo sapiens*, indicating a widespread role in gene regulation (miRBase release 18, microrna.sanger.ac.uk). miRNAs inhibit translation by binding imperfectly to sequences within the 3′ untranslated region (3′UTR) of specific messenger RNAs (mRNAs) [17], [18]. By contrast, small interfering RNAs (siRNAs), which are produced by introducing foreign dsRNAs [19] or by endogenously transcribed double-stranded RNAs (dsRNAs) [20], bind to RNA sequences with perfect complementarity leading to specific cleavage and subsequent degradation of the target mRNA. The introduction of dsRNAs or siRNAs is extensively used by many researchers to knock down the expression of mRNAs, a process called RNA interference (RNAi) [19].

Initially, most miRNAs are transcribed by RNA polymerase II as a long primary miRNA transcript (pri-miRNA) [21] which is capped and polyadenylated [22]. The pri-miRNA, which can contain clusters of miRNAs, is processed by the RNAase III enzyme Drosha [23] and the dsRNA binding protein, DGCR8 [24], into precursor miRNAs (pre-miRNAs) in the nucleus. The pre-miRNA folds into a stem loop structure and is recognized and exported to the cytoplasm by Exportin 5 [25]–[27]. The ∼70 nucleotide pre-miRNA is then processed by another RNAse III enzyme, Dicer [28]–[30], and its binding partner, TRBP [31], [32], into a mature 21–23 nucleotide dsRNA containing 2 nucleotide 5′ overhangs. The dsRNA is delivered to the RNA-inducing silencing complex (RISC), which contains an Argonaute (Ago) protein [31], [33]–[35]. One of the strands of the dsRNA (the guide strand) remains with the RISC complex; selection is determined by the most stable 5′ end of the duplex [36], [37]. The miRNA-containing RISC complex binds to the target sequence within the 3′UTR of the mRNA [38]. If the miRNA pairs with the target sequence with perfect complementarity, the target sequence undergoes endonucleolytic cleavage specifically by Ago2 and the mRNA is subsequently degraded [39]. By contrast, most miRNAs pair with imperfect complementarity leading to translational repression and/or degradation of the mRNA [38]. The mechanism by which miRNAs exert translational repression remains controversial [40]. Several models have been proposed including miRNA-directed inhibition of the cap-binding complex, inhibition of translation elongation, and deadenylation stimulation leading to subsequent degradation of target mRNA. Besides the main miRNA biogenesis pathway described, a subset of miRNAs mature via alternate pathways including Drosha-independent and Dicer-independent pathways and can originate from tRNA precursors and introns [41], [42].

The maturation of miRNAs can be regulated at the transcriptional and post-transcriptional levels and are impacted by distinct signaling pathways. Specifically, miRNA biogenesis can be regulated at distinct steps thus altering the rate at which pri-, pre- and mature miRNAs are processed. For instance, Drosha activity can be regulated positively and negatively to affect pri-miRNA maturation. Drosha-mediated processing of the pri-miRNA let-7 can be blocked by lin-28 [43]. Furthermore, estradiol stimulation can inhibit pri-miRNA processing of a subset of miRNAs by inducing estrogen receptor-α interactions with Drosha [44]. By contrast, it has been shown that TGF-β signaling pathway can promote the processing of pri- to pre-miRNA of miR-21 through Smad association with Drosha [45]. KSRP, an RNA binding protein known for its role as a splicing factor, is also a component of Dicer and Drosha complexes and is involved in the biogenesis of a subset of miRNAs [46]. Dicer activity can be regulated by the MAP/ERK kinase pathway via phosphorylation of its binding partner TRBP [47]. Finally, RISC activity can be targeted. Growth factor treatment of cells enhance the stability of Ago2, thus effectively promoting global miRNA and siRNA activities [48].

Chemical biology approaches have provided insights into the signaling pathways that control miRNA activity. Enoxacin, which was discovered via a cell-based high-throughput screen, enhances siRNA-mediated suppression of a target mRNA, and promotes miRNA biogenesis by acting at the TRBP-mediated stage [49], [50]. In other studies, a modified azobenzene compound was identified to specifically inhibit miR-21 activity by targeting pri-miRNA transcription [51] and small molecules have been found to modulate the liver-specific miR-122 levels [52]. In addition, Watashi et al. (2010) discovered two compounds, polylysine and trypaflavine, that inhibit Dicer and Argonatute activity, respectively [53]. Such studies can generate valuable tools for elucidating regulatory and signaling pathways of miRNA/siRNA function and may provide lead compounds for treatment of miRNA-associated diseases. In this study, we have used a cell-based high-throughput approach to screen for compounds from marine and plant extracts that inhibit miRNA/siRNA activity. We have identified a compound of the Daphne diterpenoid family - called genkwanine M (GENK) [54]- which affects diverse cellular functions including the inhibition of miRNA activity.

## Results

### Identification of Chemical Inhibitors of miRNA Activity

**Figure 1:**
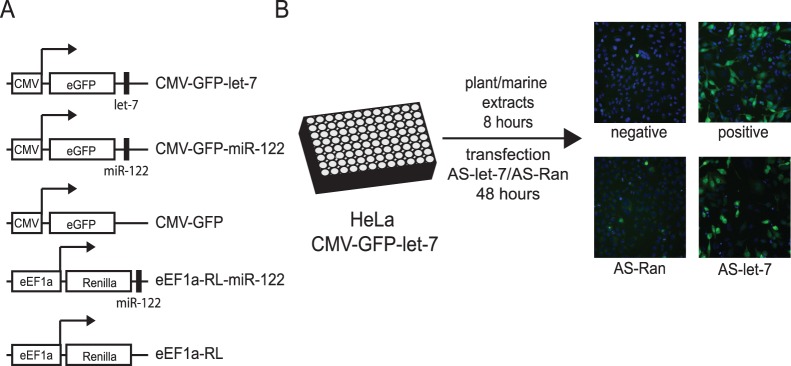
Cell-based assay for the identification of small molecule miRNA inhibitors. (A) Schematics of CMV and eEF1a promoter-driven expression plasmids that contain the eGFP or Renilla luciferase (RL) reporter gene alone or with a perfectly complementary binding site for let-7a or for miR-122 (black rectangle). (B) Stably transfected HeLa cells expressing CMV-GFP-let-7 were incubated with a series of plant or marine extracts for 8 hours or transfected with 2′ O-methyl antisense let-7 (AS-let-7) or random RNAs (AS-Ran) for 2 days. Cells were subsequently fixed and stained with Hoechst dye, and imaged by a high-throughput microscope (Cellomics). Representative images are shown of cells treated with a positive or negative extract and of cells transfected with AS-let-7 or AS-Ran.

**Figure 2:**
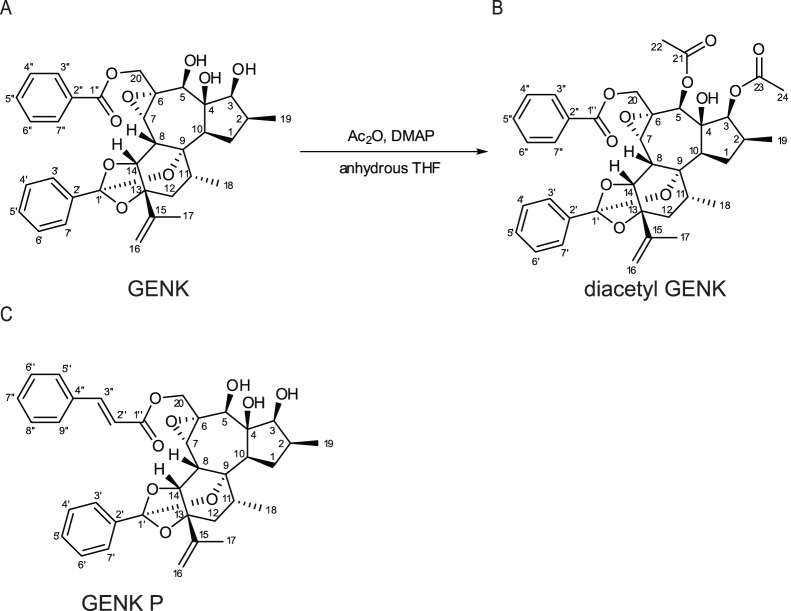
Structure of (A) genkwanine M (GENK), (B) diacetyl GENK, and (C) genkwanine P. (B) Diacetyl GENK was prepared from GENK by treatment with acetic anhydride and DMAP in anhydrous THF.

To identify compounds that interfere with miRNA and siRNA regulation and/or processing, we screened natural products using a cell-based reporter system that monitors miRNA and siRNA activity. A stably transfected clonal HeLa cell line expressing a reporter RNA encoding the *eGFP* gene and a sequence that bears perfect complementarity to the let-7a miRNA within the 3′UTR (CMV-GFP-let-7) was generated (Figure 1A). Under basal conditions, endogenously-expressed let-7a binds to the complementary sequence leading to Ago2-mediated cleavage of the reporter RNA resulting in low GFP expression (Figure 1B). By contrast, transfection of an antisense 2′-O-methyl let-7 (AS-let-7) but not a randomized antisense RNA (AS-Ran) resulted in GFP expression, indicating that GFP expression is under the control of let-7a (Figure 1B). Using this cell-based reporter system, a library of crude marine and plant extracts (∼12,000) was screened and four crude extracts were identified that led to an increase in GFP expression (Figure 1B). Specifically, we searched for extracts that led to a >3 fold increase GFP expression over background which is similar to that observed when cells were transfected with the AS-let-7. Guided by the reporter GFP system, sequential fractionation of one of the extracts led to the isolation of genkwanine M (GENK) (Figure 2A). Unfortunately, the other three extracts did not yield a pure compound. As a result, we focused on the characterization of GENK and its cellular effects.

### Isolation of GENK

The plant extract containing GENK was prepared from the leaves and twigs of a 0.5 m tall shrub of *Wikstroemia polyantha* (family Thymelaeaceae), collected on the centerline of Peninsula Malaysia approximately 50 miles south of the Thai border by E. Soepadmo and M. Suhaimi under contract with the University of Chicago at Illinois. A voucher numbered Q66O4184 was deposited in the Field Museum in Chicago and at the National Herbarium at the Smithsonian. The plant material was transferred to the NCI Open Repository where it was dried and then extracted with CH_2_Cl_2_ and MeOH. The crude extract was entered into the Open Repository screening plates as sample number NO44759. Three grams of the crude extract were supplied to UBC by D. Newman of the NCI Open Repository. Detailed procedures on the isolation and purification of GENK are described in the Materials and Methods and in the Supplementary Materials S1.

### Elucidation of GENK Structure

GENK (Figure 2A) gave a [M+Na]^+^ ion at m/z 613.2421 in the HRESIMS consistent with a molecular formula of C_34_H_38_O_9_ (calcd for C_34_H_38_O_9_Na, 613.2414), requiring 16 sites of unsaturation. The diterpenoid constitution and most of the relative configuration of GENK was determined by detailed analysis of 1D and 2D NMR data (Supplemental Materials S1). However, some of the relative configurations in the region C-1 to C-7 could not be assigned with certainty from the NMR data. Therefore, GENK (Figure 2A) was converted to its diacetyl derivative (Figure 2B) (see Supplemental Materials S1 for NMR and MS data), which gave crystals from a 9∶1 CCl_4_/hexane solution that were suitable for single crystal x-ray diffraction analysis. An ORTEP diagram that shows the constitution and absolute configuration of the diacetyl derivative is shown in Figure 2B (Supporting Materials S1).The NMR data obtained for GENK showed no evidence for the presence of acetate groups, indicating that the structure of the natural product as shown in Figure 2A, which is identical to that reported for the daphnane diterpenoid genkwanine M [54].

### GENK Effects on miRNA Activity

**Figure 3:**
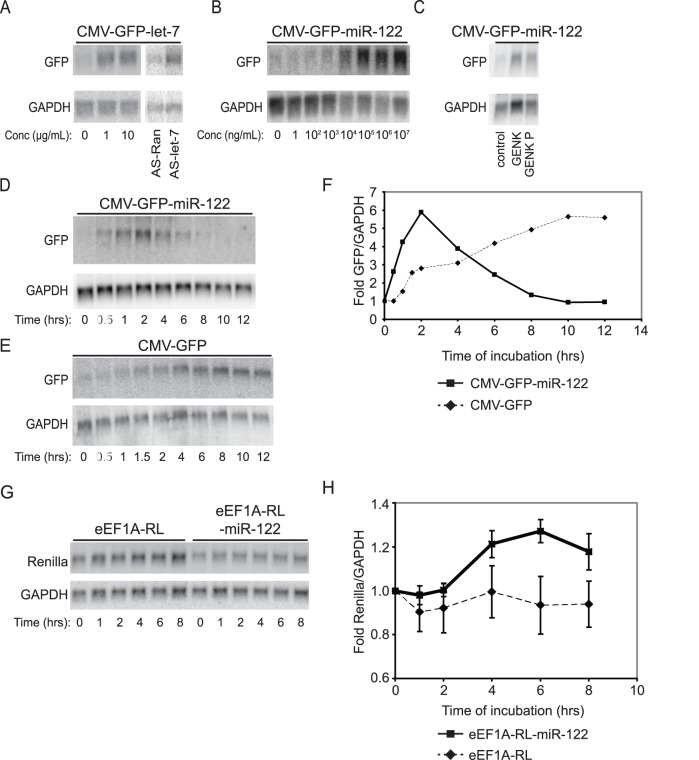
GENK effects on reporter RNA levels. (A) eGFP-let-7 mRNA levels in HeLa cells incubated with GENK for 6 hrs or transfected with AS-let-7 or AS-Ran 2′-O-methyl RNAs for 2 days. (B, C) eGFP-miR-122 mRNA levels in Huh-7 cells treated with either (B) GENK or (C) GENK P for 2 hours at the indicated concentrations (10 µM for (C)). (D, E) eGFP-miR-122 (D), eGFP (E), and GAPDH mRNA levels in Huh-7 cells treated with 10 µM of GENK for the indicated time. (F) Quantitation of eGFP-miR-122 and eGFP mRNA levels normalized to GAPDH from (D) and (E). (G) RL and RL-miR-122 reporter mRNA levels in Huh-7 cells treated with 10 µM GENK for the indicated times. (H) Quantitation of RL and RL-miR-122 mRNA levels normalized to GAPDH from (G). At 6 and 8 hours GENK treatment, the RL-miR-122 mRNA levels were ∼1.2 fold higher than the RL mRNA levels (6 hours, p-value = 0.0192; 8 hours, p-value = 0.055). For each experiment, cells were transiently transfected with the indicated reporter plasmids for 24 hours before GENK treatment. Shown are representative Northern blots from at least three independent experiments.

To confirm the effects of GENK on let-7a, CMV-GFP-let-7 reporter RNAs were monitored by Northern blot analysis. Treating CMV-GFP-let-7-expressing HeLa cells with GENK at 1 and 10 µg/mL (1.7 µM and 17 µM) increased the levels of GFP RNA by approximately two fold, similar to that observed when cells were transfected with AS-let-7 but not AS-RAN (Figure 3A). To determine whether this effect is specific to let-7 in HeLa cells, GENK was tested in Huh-7 hepatocarcinoma cells stably transfected with the CMV-GFP-miR-122 reporter. miR-122 is highly expressed in Huh-7 cells, representing ∼70% of all miRNAs expressed in this cell line [55], [56]. Similar to that observed in HeLa cells, GENK treatment resulted in an increase in GFP RNA with an EC_50_ of 8.3 µg/mL or 14 µM (Figure 3B). In addition, a minor product in the extracts, which was identified as the new daphnane diterpenoid genkwanine P (Figure 2C, Supplemental Materials S1) was determined to be 70% as active as GENK (Figure 3C) in the stably expressing CMV-GFP-miR-122 Huh-7 cell line. Treatment of HeLa and Huh-7 cells with 10 µM GENK for up to 24 or 48 hours did not significantly affect cell proliferation or viability as measured by the MTT assay (Supplementary Figure S1). Due to the consistent effects of GENK observed in the stably-transfected Huh-7 cells, we focused on this cell line for the remainder of the study.

To determine if GENK specifically inhibits siRNA activity, we tested if GENK can alter GFP mRNA levels in Huh-7 cells stably expressing a reporter RNA lacking the miR-122 binding site (CMV-GFP) (Fig 1A). Unexpectedly, GENK treatment resulted in an increase in GFP RNA levels in this cell line (Figure 3E, 3F), indicating that GENK may act at the level of transcription and not through miR-122. However, closer examination of the kinetics of the increase in reporter RNA levels during GENK treatment showed that GENK has a specific impact on the reporter mRNA containing the miR-122 binding site (Figure 3D, 3E). GENK treatment of CMV-GFP-miR-122 cells led to an increase in GFP RNA levels that peaked at 2 hours after treatment and then decreased over the next 6–8 hours (Figure 3D). By contrast, GENK treatment of CMV-GFP expressing cells resulted in a gradual increase in reporter RNA levels that continued to increase 8–10 hours after treatment (Figure 3E). Plotting the reporter mRNA levels (normalized to GAPDH mRNA levels) showed that GENK treatment stimulated the increase of CMV-GFP-miR-122 mRNA levels more quickly than that of the CMV-GFP mRNA levels, suggesting that miR-122 activity is temporally inhibited during GENK treatment (Figure 3F). To explore this further, we constructed expression plasmids containing different promoters. We selected the SV40 promoter. We constructed an SV40-Renilla and an SV40-Renilla-miR-122 expression plasmid (Supplementary Figure S2A). After 6 hours of GENK treatment in Huh-7 cells, the SV40 promoter constructs were induced 1.3 fold and 1.8 fold in the absence and presence of the miR-122 site, respectively, which is a similar induction observed with the CMV-RL construct (Supplementary Figure S2B, S2C). Thus, GENK treatment induced expression from both CMV- and SV40-driven reporter constructs. To separate the promoter and miRNA effects by GENK, we constructed an expression plasmid containing the eukaryotic elongation factor 1A (eEF1A) promoter (Figure 1A), which is predicted to direct transcription constitutively [57]. We transiently transfected either eEF1A-Renilla-miR-122 (eEF1a-RL-miR-122) or eEF1A-Renilla (eEF1a-RL) (Figure 1A) expression plasmids into Huh-7 cells and monitored reporter RNA levels after GENK treatment. In eEF1A-RL transfected Huh-7 cells, reporter RNA levels remained constant for 8 hours of 10 µM GENK treatment, demonstrating that unlike that observed with the CMV- and SV40-promoter driven reporters, GENK had minimal effects on the transcription from the eEF1A promoter (Figure 3G, 3H). For the eEF1a-RL-miR-122 construct, GENK reproducibly increased reporter RNA levels to a minor extent (1.2 fold) after 4 to 8 hours of treatment (Figure 3G, 3H). Thus, GENK can both stimulate CMV and SV40-promoter-dependent transcription and inhibit miR-122 activity.

### GENK does not Affect miRNA Levels

**Figure 4:**
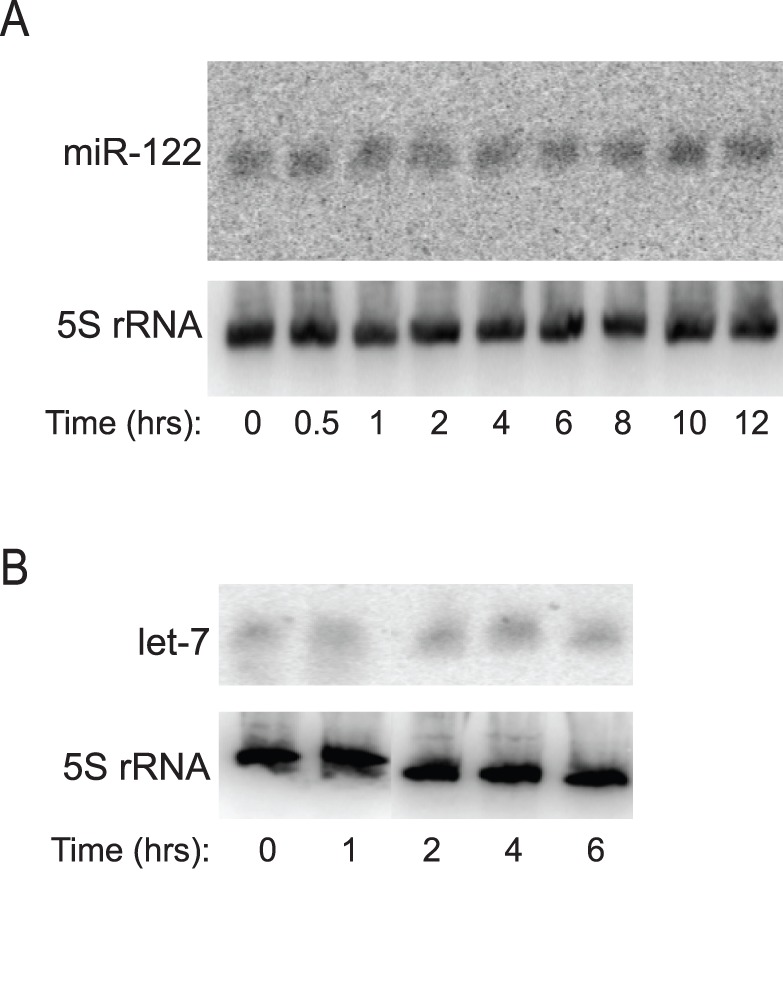
GENK effects on mature miRNA levels. Mature miR-122 levels (A) or let-7a miRNA levels (B) in Huh-7 and HeLa cells, respectively, treated with 10 µM GENK for the indicated times. Shown are representative Northern blots from at least three independent experiments.

Given that GENK inhibited miRNA activity, we explored the possibility that it might affect miRNA levels. Total RNA from cells treated with GENK was isolated and loaded on a high percentage acrylamide gel to resolve miRNAs [58]. Northern blotting showed that mature miR-122 levels in Huh-7 cells (Figure 4A) and mature let-7a levels in HeLa cells (Figure 4B) were not affected during GENK treatment, suggesting that GENK may affect miRNA activity directly rather than the transcription or stability of miRNAs. To investigate this, we tested whether GENK can inhibit miRNA-dependent cleavage in an *in vitro* assay. Towards this, an *in vitro* transcribed reporter RNA, containing a perfectly complementary site for miR-451 or a miR-451 site in the reverse complement direction, was incubated in rabbit reticulocyte lysates (RRL) (Supplementary Figure S3A). miR-451 is highly expressed in RRL [59]. Specific cleavage of the reporter RNA (388 nucleotides) directed by miR-451 resulted in a 322 nucleotide RNA fragment (Supplementary Figure S3A). Incubation of the 5′-[^32^P]-labeled reporter RNA containing the complementary miR-451 but not the reverse complement miR-451 sequence resulted in specific cleavage of the reporter RNA (Supplementary Figure S3B, 10 minute incubation). Addition of 10 µM GENK to the extracts did not alter the rate of cleavage of the reporter RNA (Supplementary Figure S3B). Several conditions were tested in which GENK was preincubated with the extract prior to addition of the reporter RNA and GENK was incubated at different concentrations and for longer times, none of which altered the cleavage rate (data not shown). In summary, these data suggests that GENK treatment does not inhibit the maturation of miRNA or affect miRNA or siRNA activity as monitored by an *in vitro* cleavage assay.

### GENK Induces an Inflammatory Response

**Figure 5:**
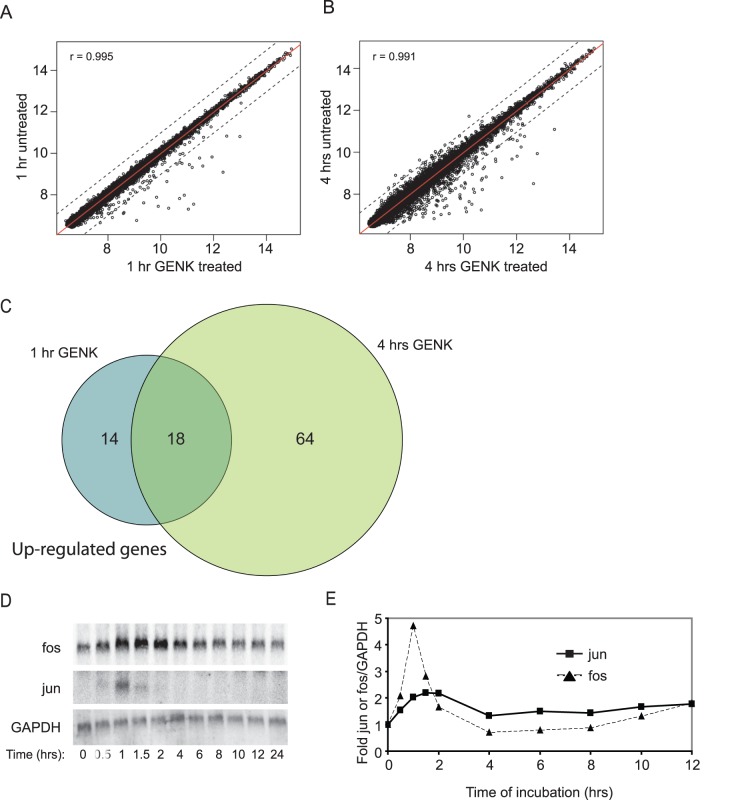
Transcriptional response elicited by GENK treatment. (A and B) Scatter plots of microarray-derived mRNA expression in Huh-7 cells treated with DMSO or 10 µM GENK for (A) 1 or (B) 4 hours. At the indicated times, total mRNA was isolated and analyzed as described in the Experimental Section. Normalized relative hybridization signal intensities of GENK treatment (x-axis) versus DMSO treatment (y-axis) are plotted. The data values are highly correlated (Pearson correlation 0.995 for 1 hour treatment and 0.991 for 4 hours treatment). Dotted black lines correspond to 2-fold change (up or down-regulation). (C) Venn diagram of genes up-regulated at least 2-fold after 1 and 4 hours GENK treatment. (D) Fos and Jun mRNA levels in Huh-7 cells treated with 10 µM GENK for the given times are shown by northern blot analysis. (E) Quantitation of mRNA levels from (D) normalized to GAPDH mRNA levels.

The observation that GENK treatment stimulated CMV transcription, but not eEF1A-driven transcription suggests GENK may stimulate transcription of a subset of genes. To explore this further, we profiled the transcriptome of Huh-7 cells treated with GENK for 1 and 4 hours using microarray analysis. GENK induced the expression of 0.07% (32 mRNAs) and 0.17% (94 mRNAs) of total mRNAs by 2-fold or more at 1 and 4 hours GENK treatment respectively (Figure 5A, 5B) (p-value <0.01, BH-adjusted). Based on the two time points, several trends in gene expression are observed. One, a significant subset of mRNAs (23%) are up-regulated at both 1 and 4 hours GENK treatment (Supplementary Figure S4). Second, a small number of mRNAs were significantly down-regulated in GENK-treated cells (13 mRNAs at 4 hours) (Supplementary Figure S4). Third, the majority of mRNAs did not change significantly during GENK treatment (<2 fold). Supplementary Tables S1 and S2 display the top mRNAs that are up or down-regulated by more than 2 fold under GENK treatment for 1 and 4 hours, respectively. Supplementary Table S3 displays the top mRNAs that are up-regulated more than 2 fold at both 1 and 4 hours GENK treatment. The majority of genes include chemokines/cytokines, proteins involved in signal transduction pathways, and transcription factors or modulators. The genes induced after 1 hour GENK treatment closely matched the chemokines and cytokines induced by TNFα stimulation in 3T3 fibroblasts in a study published by Hao and Baltimore (2009) (Supplementary Table S1 and S2) [60]. Furthermore, classic early immediate genes described in that study such as c-*Fos*, c-*Jun*, *Irf1*, *Cscl2* and *Ier3* [60] were induced during GENK treatment. Thus, the results indicate that GENK treatment induces an early inflammatory response.

To confirm these results, we monitored Fos and Jun mRNA levels during GENK treatment. Unlike GAPDH mRNA levels, Fos and Jun (Figure 5D, 5E) mRNA levels were transiently up-regulated within the first few hours of GENK treatment before decreasing at later time points, in agreement with previous reports and the microarray analysis, and consistent with the idea that GENK treatment induces the early inflammatory response [60]. The transient induction of Fos and Jun (Figure 5D, 5E) mRNA levels is similar to the trend observed with the induction of the reporter CMV-GFP-miR-122 mRNA levels (Figure 3D, 3F).

### TNFα Inhibits miR-122 Activity

**Figure 6:**
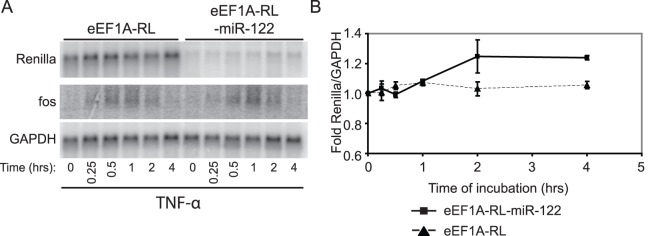
Treatment of cells with TNFα. (A) Northern blot analysis of reporter Renilla luciferase (RL) and RL-miR-122 mRNA levels in Huh-7 cells treated with 10 ng/mL of TNFα for the indicated times. Huh-7 cells were transiently transfected with eEF1A-RL or eEF1A-RL-122 plasmids for 24 hours before TNFα treatment. (B) Quantitation from (A) of at least three independent experiments. At 4 hours TNFα treatment, the RL-miR-122 mRNA levels were ∼1.2 fold higher than the RL mRNA levels (p-value = 0.012).

Given that GENK treatment activated the inflammatory response and also inhibited miR-122 and let-7 miRNA, we investigated whether a well-established inducer of the inflammatory response, TNFα, has an effect on miRNA inhibition. Huh-7 cells were transiently transfected with eEF1a-RL and eEF1a-RL-122 and then treated with 10 ng/mL TNFα. Under this treatment, the reporter RNA increased to the same degree and had similar kinetics as that observed with cells treated with GENK (Figure 6). As shown previously, TNFα treatment resulted in an increase in Fos mRNA [60]. Thus, these results suggest that GENK and TNFα activate a similar response, which includes a moderate inhibition of miRNA activity and induction of CMV-driven transcription (Figure 6).

### GENK Treatment Inhibits HCV Infection

**Figure 7:**
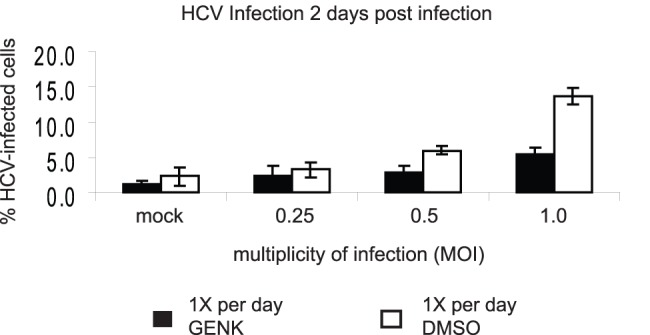
Inhibition of HCV replication in Huh-7 cells by GENK. Huh-7 cells were mock-infected or infected with HCV at a MOI of 0.25, 0.5, or 1 in the presence of DMSO or 10 µM GENK. DMSO or GENK was added 24 hrs prior to infection and subsequently every 24 hrs. 48 hrs post-infection, cells were fixed then probed with an HCV-core protein specific antibody and cell nuclei were stained with Hoechst dye. The percentage of HCV infected cells (positive for core expression) was determined by immunofluorescence using a high-throughput microscope (Cellomics). At a MOI of 0.5 and 1, 10 µM GENK treatment inhibited HCV infection as compared to DMSO treatment (p-value <0.0001). There was no difference in the percentage of cells with immunofluorescence above background in mock-infected or HCV-infected cells (MOI 0.25) treated with DMSO or 10 µM GENK (p-value = 0.2918 and p-value = 0.2484, respectively).

miR-122 is necessary for Hepatitis C virus (HCV) replication in Huh-7 cells [14]. Given our observations that GENK induces the inflammatory response and moderately inhibits miR-122 activity, we tested whether GENK may inhibit HCV replication. Infection of Huh-7 cells by the infectious HCV clone, JFH-1, at low multiplicity of infection (MOI of 0.25 to 1.0) resulted in ∼4–15% of the cells showing fluorescence by antibody staining to the HCV core protein at 2 day post-infection (Figure 7). By contrast, addition of GENK significantly decreased the percent of cells infected by HCV. At an MOI of 1, GENK inhibited HCV infection by approximately 3 fold (Figure 7). Thus, GENK treatment can inhibit HCV infection in Huh-7 cells.

## Discussion

MiRNAs play important roles in cellular homeostasis, development, and disease. Despite the discovery of the main factors involved in miRNA biogenesis, the regulation and signaling pathways that impact miRNA maturation and function are poorly understood. In this study, we have used a miRNA-sensitive reporter cell line to identify a small molecule from a collection of plant and marine extracts that induces the early inflammatory response and moderately inhibits miRNA function. Using reporter constructs, we demonstrated that GENK inhibits miRNA-mediated repression in a time-dependent manner, thus suggesting that tight control of miRNA activity is involved in the early inflammatory response.

Gene profiling revealed that GENK treatment of Huh-7 cells induced an early inflammatory response as several signature markers such as Fos and Jun mRNA levels are increased early during GENK treatment of cells (Supplementary Table S1 and S2, Figure 5). The expression profiles of GENK-treated cells are remarkably similar to those of TNFα treated cells. It has been shown that the early inflammatory response mediated by cytokines such as TNFα is regulated by transcription factors such as NF-κB, AP-1 and interferon-regulatory factors [60]. The CMV and SV40 promoters contain binding sites for the transcription factors NF-κb and AP-1 [61], [62]. Thus, the activation of the CMV and SV40-driven reporter constructs by GENK treatment in our studies is likely due to the induction of an inflammatory response via NF-κb and AP-1 signaling.

Both TNFα and GENK treatments lead to a rapid increase of Fos and Jun mRNAs followed by rapid degradation (Figure 5) [60]. This early burst of expression is mediated by several mechanisms including transcriptional induction and increased mRNA stability through AU-rich elements (AREs) in the 3′UTR. Early immediate genes such as Fos and Jun contain 3′UTR AREs which destabilize mRNAs under basal conditions. In response to external signals such as ionizing radiation or immune signaling, ARE-mediated decay is inhibited for a short time to allow stabilization of and translation of ARE-containing mRNAs [60]. Prolonged or chronic misregulation of this posttranscriptional response can lead to inflammatory disorders and disease. For example, targeted deletion of the ARE in TNFα in mice leads to chronic inflammatory arthritis and Crohn’s-like inflammatory bowel disease [63]. Interestingly, a previous study reported that the miRNA pathway may play a role in regulating the stability of AREs and that TNFα treated cells can repress siRNA-mediated RNA interference [8], [64]. In our study, we have shown that miRNA-dependent repression can be inhibited by GENK treatment, albeit to moderate levels. The level of repression was reproducible and consistently ∼1.2 fold using reporter constructs under the control of the constitutive eEF1A promoter (Figure 3G, 3H). Although the repression was moderate, there was a significant impact on the kinetics of expression of reporter constructs driven by the CMV promoter. In the miRNA-regulated reporter CMV-GFP-miR-122 construct, the reporter RNA levels peaked at 2 hours before decreasing (Figure 3D, 3F), which is a pattern similar to that observed with other classic early response genes such as Fos and Jun during an inflammatory response (Figure 5D) [60]. By contrast, the CMV-GFP reporter RNA lacking a miRNA binding site showed a gradual increase in expression over time (Figure 3E, 3F). This strongly indicates that miR-122-mediated repression of the CMV-driven reporter construct is inhibited during GENK treatment to allow for the rapid increase in reporter mRNA levels. These results support the idea that the combination of miRNA-mediated repression, albeit minor, and transcriptional induction during GENK treatment contribute to the tight regulation of genes during the inflammatory response, possibly those mRNAs containing AREs.

GENK does not alter mature let-7a or miR-122 levels, suggesting that the inhibition of miRNA activity is likely at the level of RISC activity (Figure 4). GENK does not affect siRNA-mediated cleavage in an *in vitro* cleavage assay. How GENK inhibits miRNA/siRNA activities *in vivo* remains to be investigated. Given the pleiotropic effects of GENK, it seems likely that inhibition of miRNA activity is a result of the induction of the inflammatory response.

GENK inhibits HCV virus infection in Huh-7 cells (Figure 7), which is likely due to activation of an inflammatory response and to a minor extent inhibition of miR-122 activity by GENK. Previously, it has been reported that HCV replication is resistant to TNFα [65]. Thus, despite similarities of the effects tested in this study by GENK and TNFα, GENK may be inhibiting HCV virus infection specifically and distinct of the effects observed by TNFα.

Chemical biology approaches provide an alternative to genetic screens in identifying signaling pathways that affect major biological processes. The use of reporter constructs that sense miRNA or siRNA activity has been extremely useful for identifying chemical inhibitors of this pathway [49]–[52]. Although the CMV and SV40 promoters are strong transcriptional promoters and have been used widely for expression studies, these promoters are highly inducible especially during signaling events that activate an inflammatory response [57], [61], [62]. The use of a constitutive promoter such as the eIF1A promoter used in this study should be useful for future screenings of chemical inhibitors of miRNA function.

## Methods

### Plasmids

CMV-GFP-let-7 and CMV-eGFP-miR-122 were constructed by inserting a perfectly complementary let-7 or miR-122 binding site within an MfeI restriction enzyme site between the eGFP and SV40 poly A signal in pEGFP-N1 vector (Clontech). eEF1A-RL and eEF1A-RL-miR-122 were constructed by swapping the CMV promoter of CMV-GFP-let-7 or CMV-GFP-miR-122 with the eEF1A promoter from pTy-eGFP (kindly provided by Ivan Sadowski, University of British Columbia) using the restriction site BglII and HindIII. eEF1a-RL-122 was constructed by PCR with primers containing an Nhe1 site with the initial ORF of RL and a primer containing the end of the reverse complement ORF of RL, miR-122 binding site and XbaI site. This fragment was subsequently swapped into the eEF1a-RL, replacing the RL with NheI and XbaI restriction sites. SV40-RL (provided by Martin Bushell, University of Notingham) was used as a template to construct SV40-RL-miR122 and CMV-RL. Specifically, SV40-RL-miR122 was constructed by introducing miR122 into the Xba1 site. And CMV-RL was made by swapping in SV40 promoter with full length CMV promoter (from pCDNA3, Invitrogen) using BglII and HindIII restriction sites.

### Cell Culture and Stable Cell Lines

HeLa and Huh-7 cells were grown in Dulbecco’s modified Eagle medium (DMEM) (Sigma) with 10% fetal bovine serum (v/v) (Hiclone), 2 mM L-glutamine (Invitrogen), and 50 µg/mL penicillin/streptomycin (Invitrogen). HeLa and Huh-7 cells were obtained by stocks held at Stanford University, Stanford, California, from previous studies [14], [66]. Stable cell lines were selected by transfection with Lipofectamine 2000™ (Invitrogen) and using 1 mg/mL Geneticin (Invitrogen). All of the cells were maintained at 37°C in 5% CO_2_.

### Screen for miRNA Inhibitors

HeLa cells stably expressing EGFP-let-7 by CMV promoter were seeded in PerkinElmer View 96-well plates at 8,000 cells per well in 100 µL DMEM at 37°C. 24 hours after seeding, 340 nL of marine and plant extracts were added to each well using a Biorobotics Biogrid II equipped with a 0.7 mm diameter 96-pin tool. Plates were incubated for 8 hours at 37°C, the medium was aspirated and cells were fixed with 3% paraformaldye in PBS for 15 min at room temperature. After two washes with PBS, the cells were incubated with 500 ng/mL Hoechst 33342 for 15 min at room temperature. Plates were read in a Cellomics™Arrayscan VTI automated fluorescence imager. Cells were photographed with a 10× objective in the Hoechst and FITC (XF-93 filter) channels. 10 images per well were collected. The target activation analysis algorithm was used to identify cells via Hoechst stain of the nuclei (ObjectAreaCh1 = 5) in the first channel and apply a cytoplasmic mask in the second FITC channel around the nucleus. This allows us to determine the average cytoplasmic GFP intensity of each cell. The average GFP intensity of all the cells in each well was computed at fixed 200 pixel intensity units. Guided by the AS-let-7 transfection experiments, we determined that a 3 fold increase in GFP expression in cells over that observed in untreated cells is the minimal threshold to identify a positive candidate extract. Wells that showed less than 4,000 cells were discarded as toxic extracts. 12,000 plant and marine extracts from the NCI open repository were screened using this protocol.

### Isolation of GENK

To Isolate GENK, three grams of the crude extract were partitioned between EtOAc (3×50 mL) and H_2_O (100 mL). The combined EtOAc extract was evaporated to dryness to give 2.1 g of dark green oil; 1.00 g of this was chromatographed on a 10 g normal phase Waters Sep-PakPak, employing a step gradient from 95∶5 *n*-Hexanes/EtOAc to EtOAc, and from 90∶10 EtOAc/MeOH to MeOH, to give fractions A-E. Fraction C (38 mg) eluting with 60∶40 *n*-Hexanes/EtOAc, was chromatographed on a Sephadex® LH-20 column using 1∶1 MeOH/CH_2_Cl_2_ as an eluent to give fractions C-(A-G). Pure GENK (Figure 2A) (4.9 mg) and a minor product, genkwanine P (Figure 2C) (2.1 mg) were obtained aswhite amorphous solids from fraction C-E (15 mg) via C-18 reversed-phase HPLC using 8∶2 MeOH/H_2_O as an eluent over 70 min. (flow rate 2 mL/min). A more detailed protocol can be found in the Supplementary Materials S1.

### Northern Blot Analysis

Total RNA from cells was purified using TRIzol (Invitrogen). 5 µg of each sample was loaded onto a 1% denaturing agarose gel (1× MOPS and 20% formaldehyde v/v). RNA was transferred and crosslinked to Zeta-probe membrane (Bio-Rad, UV Stratalinker 1800™ by Strategene). Transfer of RNA was confirmed by methylene-blue staining. The membrane was pre-hybridized with 5 mL hybridization buffer (0.5 M NaPO_4_, 10 mM EDTA, 7% SDS w/v) at 65°C for at least 30 min. [α-^32^P] dATP-labeled DNA probes were generated using the Radprimer kit (Invitrogen) and subsequently purified using the QIAquick Nucleotide Removal Kit (Qiagen). For miRNA detection, 20 µg of RNA was loaded onto a 15% denaturing acrylamide gel and subsequently transferred to a nitrocellulose membrane by a Trans-Blot SD Semi-Dry Transfer Cell™ (BioRad) for 1 hr at 12 V. After transfer, RNAs were UV-crosslinked to the membrane (UV Stratalinker 1800™ by Strategene). 5′ end-labeled LNA antisense miR-122 or let-7 oligos (Exiqon) were used for Northern blot analysis. Probes were heated at 95°C before adding to the pre-hybridized membranes. After incubating overnight at 65°C, the membranes were washed three times with 15 mL of Wash Buffer (0.1% SDS w/v, 0.1× SSC) at 50°C. The membranes were analyzed by phosphorimager analysis (Typhoon, GE Healthcare).

### *In vitro* siRNA Cleavage Assay in Rabbit Reticulocyte Lysate

The miR-451 sensor was constructed by PCR amplification using primers that amplified the RL gene with the miR-451 sequence in the sense or antisense direction. The PCR product was subsequently cloned into the pCRII-TOPO vector (Invitrogen). The reporter miR-451 RNA was *in vitro* transcribed by a T7 RNA polymerase reaction. The reporter RNA was dephosphorylated and then 5′ end-labeled with [γ-^32^P]-ATP by T4 polynucleotide kinase (Fermentas). The RNAs were purified using an RNeasy Kit (Qiagen). Radiolabel RNAs (500,000 cpm) were incubated in Rabbit Reticulocyte Lysates (Promega) pretreated with or without 10 µM GENK and aliquots of the reaction were taken at different times. RNA was extracted from the samples by phenol/chloroform extraction and ethanol precipitation. Equal cpm of RNA were loaded on a 12% acrylamide gel, dried, and analyzed by phosphorimager analysis.

### Microarray Analysis

Total RNA extracted from Huh-7 cells pretreated with DMSO or 10 µM GENK for 1 and 4 hours and performed in triplicate. Illumina Direct Hybridization assays were performed by the Vancouver Prostate Centre Laboratory for Advanced Genome Analysis, Vancouver, Canada. Total RNA quality was assessed with the Agilent 2100 bioanalyzer prior to microarray analysis. Samples with a RIN value of greater than or equal to 8.0 were deemed to be acceptable for microarray analysis. An input of 200 ng of total RNA was used to generate biotin-labeled cRNA following the Illumina® TotalPrep RNA Amplification Kit (Ambion, Inc.). Samples were hybridized on Illumina HumanHT-12 v3 BeadChips following the Illumina Whole-Genome Gene Expression Direct Hybridization Assay Guide (11286331). BeadChips were imaged and quantified with the Illumina iScan scanner and data was processed with Illumina BeadStudio v3.3.8. Data processing included averaging signal intensities for each unique Beadtype. The microarray data has been deposited to GEO with accession number: (will be submitted).

Differential expression analysis was carried out between samples treated with drug for 1 hr and 4 hours relative to untreated control. log_2_ normalized expression values were used for statistical analysis using the *limma* package in R. The p-values were adjusted for multiple correction using the Benjamini-Hochberg algorithm. A stringent criteria consisting of fold change of 2 (up/down-regulation) and an adjusted *p-*alue of 0.01 was used to determine significantly deregulated mRNAs. Clustering of differentially expressed genes from both treatment groups was carried out using complete linkage hierarchical clustering with Euclidean distances with the *gplots* package in R. Pathway analysis was performed using the Ingenuity Pathway database. A p-value of 0.05 (-log(*p*) of 1.30) was used to determine significantly enriched pathways.

### Detection of HCV-infected Cells

A cDNA copy of HCV JFH-1 (a gift from Dr. Takaji Wakita, National Institute of Infectious Diseases, Tokyo, Japan) was *in vitro* transcribed to genomic RNA which was then used to produce infectious virus stocks as previously described [67], [68]. Filtered HCV JFH-1 stocks were used to infect Huh-7 cells pretreated with 10 µM GENK for 24 hours. At 2 days post-infection, cells were fixed with 4% v/v formaldehyde in PBS, washed with PBS and then permeabilized and blocked with PBS containing 3% BSA, 0.3% Triton X and 10% FBS (Blocking buffer) for 1 hr. Cells were then probed with mouse anti-core C7-50 primary antibody (1∶500, Abcam) in Binding Buffer (Blocking buffer without FBS) at 4°C overnight then washed 3 times with PBS. Cells were probed with diluted donkey anti-mouse Alexa-Fluor-568 conjugated secondary antibody (1∶1000) and 10 µg/ml Hoecsht 33258 in binding Buffer for 1 hr at room temperature. After three washes with PBS, cells were left in 50 µl PBS and the percentage of HCV infected cells was analyzed using the Cellomics™Arrayscan VTI automated fluorescence imager.

## Supporting Information

Figure S1 MTT assay of HeLa and Huh-7 cells treated with 10 µM GENK for the indicated times. Viability of HeLa and Huh-7 cells (100 µL volume) was monitored by the MTT colorimetric assay (the addition of final 1.25 mg/mL MTT for 2 hours). Subsequently, cells were lysed with extraction buffer (20% w/v SDS, 50% DMF, 2.5% acetic acid, and 2.5% 1M HCL) overnight. Reduction of MTT was detected at 570 nm.
(EPS)

Figure S2 GENK induces SV40 promoter expression of Renilla luciferase gene. (A) Schematics of SV40 and CMV promoter-driven expression plasmids that contain Renilla luciferase (RL) reporter gene alone or with a perfectly complementary binding site for miR-122 (black rectangle). (B) Northern blot analysis of Huh7 cells transiently transfected with plasmids containing SV40-RL, SV40-RL-miR122 or CMV-RL incubated with or without 10 µM GENK for 6 hours. (C) Quantitation of RL and RL-miR-122 mRNA levels normalized to actin in (B). Shown are the averages from two independent experiments.
(EPS)

Figure S3 GENK effects on siRNA activity using an *in vitro* cleavage assay. (A) A fragment of Renilla luciferase was engineered with a perfectly complementary binding site for miR-451 in the sense or antisense direction. (B) The miR-451 sensor RNA was *in vitro* transcribed as shown and 5′end labeled with P^32^ using a T4 kinase assay. The sensor RNA was incubated in rabbit reticulocyte lysate (RRL) (Promega) pretreated with DMSO or 10 µM GENK for the indicated times. Endogenous miR-451 in RRL binds to the miR-451 site leading to cleavage of the *in vitro* transcribed RNA to generate two fragments, 322 and 66 nucleotides in length. Only the 322 nucleotide fragment will be detected by a phosphorimager. The RNAs were extracted and separated on a 12% acrylamide gel, which was dried and detected by a phosphorimager analysis.
(EPS)

Figure S4 Clustering of differentially expressed mRNAs from 1 and 4 hours treatment groups. Heatmap showing mRNAs that are differentially expressed at 1 and 4 hours GENK post treatment. Each mRNA needs to be significantly deregulated in at least one treatment group to be included. Colors indicate absolute fold changes according to the specified scale. Red denotes up-regulation relative to control while blue indicates down-regulation. Clustering was carried out using the complete linkage clustering algorithm. Whereas a small number of mRNAs are down-regulated at 4 hours after treatment, the majority are up-regulated.
(EPS)

Table S1 **List of genes up-regulated at 1 hr treatment with GENK** (fold change threshold of 2, adjusted p-value of 0.01).
(DOCX)

Table S2 **List of genes up-regulated or down-regulated at 4 hrs treatment with GENK** (fold change threshold of 2, adjusted p-value of 0.01).
(DOCX)

Table S3 **List of genes up-regulated at both 1 and 4 hours treatment with GENK** (fold change threshold of 2, adjusted p-value of 0.01).
(DOCX)

Materials S1 (DOC)
