# Supplementary material for: A Daphnane Diterpenoid Isolated from Wikstroemia polyantha Induces an Inflammatory Response and Modulates miRNA Activity
Source: PLoS One. 2012 Jun 26;7(6):e39621. doi: 10.1371/journal.pone.0039621 (PMC3383676; doi:10.1371/journal.pone.0039621)
Supplement: Table S1 — List of genes up-regulated at 1 hr treatment with GENK (fold change threshold of 2, adjusted p-value of 0.01). (DOCX) [file pone.0039621.s005.docx]

| **Table S1** | | | | |
| --- | --- | --- | --- | --- |
|  |  |  |  |  |
| **Gene name** | **Alias or gene description** |  |  | **GENK treatment**  **(fold change)** |
|  |  |  |  |  |
| **Cell death, survival** |  |  |  |  |
| C8ORF4 | chromosome 8 open reading frame 4 |  |  | 3.34 |
| PHLDA1 | pleckstrin homology-like domain |  |  | 3.03 |
|  |  |  |  |  |
| **Chemokine or cytokine** |  |  |  |  |
| CXCL2 | GRO2 |  |  | 13.37 |
| IL8 | interleukin 8 |  |  | 9.68 |
| CXCL1 | Gro1 |  |  | 5.49 |
| CCL20 | MIP3A |  |  | 3.18 |
| TNF | tumor necrosis factor |  |  | 2.08 |
| CXCL10 | IP-10 |  |  | 2.01 |
|  |  |  |  |  |
| **Signal Transduction** |  |  |  |  |
| TNFAIP3 | A20 |  |  | 7.7 |
| IER3 | immediate early response 3 |  |  | 7.52 |
| DUSP1 | MKP1 |  |  | 4.62 |
| SGK | serum/glucocorticoid regulated kinase 1 |  |  | 3.25 |
| PPP1R15A | GADD34 |  |  | 3.09 |
| MAP3K8 | mitogen-activated protein 3 kinase 8 |  |  | 2.01 |
|  |  |  |  |  |
| **Transcription factors or modulators** | |  |  |  |
| EGR1 | early growth response 1 |  |  | 12.95 |
| RASD1 | dexamethasone-induced Ras-related protein |  |  | 7.44 |
| c-FOS | FBJ osteosarcoma oncogene |  |  | 5.01 |
| NFKBIA | IkBa |  |  | 4.56 |
| c-Jun | Jun oncogene |  |  | 4.34 |
| NFKBIZ | IkBzeta |  |  | 3.66 |
| FOSB | FBJ osteosarcoma oncogene homolog B |  |  | 2.8 |
| ATF3 | activating transcription factor 3 |  |  | 2.75 |
| KLF6 | Kruppel-like factor 6 |  |  | 2.45 |
| JUNB | Jun-B oncogene |  |  | 2.41 |
| IRF1 | interferon regulatory factor 1 |  |  | 2.09 |
| ELF3 | Epithelial-restricted with serine box |  |  | 2.01 |
|  |  |  |  |  |
| **Others** |  |  |  |  |
| ZFP36 | TTP |  |  | 3.89 |
| CYR61 | cysteine-rich, angiogenic inducer, 61 |  |  | 2.27 |
| ADM | adrenomedullin |  |  | 2.23 |
| EDN1 | endothelin 1 |  |  | 2.21 |
| GDF15 | growth differentiation factor 15 |  |  | 2.2 |
| BMP2 | bone morphogenetic protein 2 |  |  | 2.16 |
|  |  |  |  |  |
