# Supplementary material for: A Daphnane Diterpenoid Isolated from Wikstroemia polyantha Induces an Inflammatory Response and Modulates miRNA Activity
Source: PLoS One. 2012 Jun 26;7(6):e39621. doi: 10.1371/journal.pone.0039621 (PMC3383676; doi:10.1371/journal.pone.0039621)
Supplement: Table S2 — List of genes up-regulated or down-regulated at 4 hrs treatment with GENK (fold change threshold of 2, adjusted p-value of 0.01). (DOCX) [file pone.0039621.s006.docx]

| **Table S2** | | | | | | | | | | | | | |  |
| --- | --- | --- | --- | --- | --- | --- | --- | --- | --- | --- | --- | --- | --- | --- |
|  | | |  | | | |  | | |  | | |  |  |
| **Gene name** | | | **Alias or gene description** | | | |  | | |  | | | **GENK treatment**  **(fold change)** |  |
|  | | |  | | | |  | | |  | | |  |  |
| **Cell adhesion** | | |  | | | |  | | |  | | |  |  |
| THBS1 | | | thrombospondin 1 | | | |  | | |  | | | 3.79 |  |
| GJA1 | | | gap junction protein | | | |  | | |  | | | 3.16 |  |
| PPAP2B | | | lipid phosphate phosphohydrolase 3 | | | |  | | |  | | | 2.92 |  |
| NLF2 | | | C2 calcium-dependent domain B2 | | | |  | | |  | | | 2.09 |  |
|  | | |  | | | |  | | |  | | |  |  |
| **Cell cycle** | | |  | | | |  | | |  | | |  |  |
| CDKN1A | | | CIP1 | | | |  | | |  | | | 2.24 |  |
| C13ORF15 | | | chromosome 13 open reading frame 15 | | | |  | | |  | | | 2.02 |  |
|  | | |  | | | |  | | |  | | |  |  |
| **Cell death, survival** | | |  | | | |  | | |  | | |  |  |
| C8ORF4 | | | chromosome 8 open reading frame 4 | | | |  | | |  | | | 6.96 |  |
| PHLDA1 | | | pleckstrin homology-like domain | | | |  | | |  | | | 5.63 |  |
| TNFRSF11B | | | Osteoclastogenesis inhibitory factor | | | |  | | |  | | | 5.05 |  |
| UBD | | | ubiquitin D | | | |  | | |  | | | 4.45 |  |
| BIRC3 | | | Apoptosis inhibitor 2 | | | |  | | |  | | | 3.81 |  |
| TNFRSF12A | | | FN14 | | | |  | | |  | | | 2.88 |  |
| DDIT4 | | | DNA-damage-inducible transcript 4 | | | |  | | |  | | | 2.67 |  |
| TNFRSF10B | | | apoptosis inducing receptor TRAIL-R2 | | | |  | | |  | | | 2.65 |  |
| PMAIP1 | | | PMA-induced protein 1 | | | |  | | |  | | | 2.62 |  |
|  | | |  | | | |  | | |  | | |  |  |
| **Chemokine or cytokine** | | |  | | | |  | | |  | | |  |  |
| IL8 | | | interleukin 8 | | | |  | | |  | | | 17.54 |  |
| CXCL10 | | | IP-10 | | | |  | | |  | | | 10.75 |  |
| CXCL1 | | | Gro1 | | | |  | | |  | | | 7.75 |  |
| CXCL6 | | | CKA-3 | | | |  | | |  | | | 5.94 |  |
| CXCL2 | | | GRO2 | | | |  | | |  | | | 5.26 |  |
| CCL20 | | | MIP3A | | | |  | | |  | | | 5.07 |  |
| CXCL5 | | | SCYB5 | | | |  | | |  | | | 4.63 |  |
| CX3CL1 | | | NTN | | | |  | | |  | | | 2.03 |  |
|  | | |  | | | |  | | |  | | |  |  |
| **Innate immunity** | | |  | | | |  | | |  | | |  |  |
| HAMP | | | hepcidin antimicrobial peptide | | | |  | | |  | | | 2.96 |  |
| PI3 | | | peptidase inhibitor 3 | | | |  | | |  | | | 2.46 |  |
| LCN2 | | | lipocalin 2 | | | |  | | |  | | | 2.19 |  |
|  | | |  | | | |  | | |  | | |  |  |
| **Receptor** | | |  | | | |  | | |  | | |  |  |
| OLR1 | | | oxidized low density lipoprotein receptor 1 | | | |  | | |  | | | 4.19 |  |
| SDC4 | | | syndecan 4 | | | |  | | |  | | | 2.9 |  |
| LDLR | | | low density lipoprotein receptor | | | |  | | |  | | | 2.13 |  |
| EPHA1 | | | EPH receptor A1 | | | |  | | |  | | | -2.17 |  |
| GPER | | | G protein-coupled estrogen receptor 1 | | | |  | | |  | | | -2.43 |  |
|  | | |  | | | |  | | |  | | |  |  |
|  | | | | | | | | | | | | | | |
|  | |  | | |  | | | | | |  | |  |  |
| **Gene name** | | **Alias or gene description** | | |  | | | | | |  | | **GENK treatment**  **(fold change)** |  |
|  | |  | | |  | | | | | |  | |  |  |
| **Signal Transduction** | |  | | |  | | | | | |  | |  |  |
| TNFAIP3 | | A20 | | |  | | | | | |  | | 6.74 |  |
| IER3 | | immediate early response 3 | | |  | | | | | |  | | 4.39 |  |
| IRAK2 | | interleukin-1 receptor-associated kinase 2 | | |  | | | | | |  | | 4.03 |  |
| DUSP5 | | dual specificity phosphatase 5 | | |  | | | | | |  | | 3.88 |  |
| LRG1 | | leucine-rich alpha-2-glycoprotein 1 | | |  | | | | | |  | | 3.72 |  |
| TRIB1 | | tribbles homolog 1 (Drosophila) | | |  | | | | | |  | | 2.88 |  |
| IFNGR2 | | interferon gamma receptor 2 | | |  | | | | | |  | | 2.82 |  |
| SGK | | serum/glucocorticoid regulated kinase 1 | | |  | | | | | |  | | 2.79 |  |
| SH2B3 | | Signal transduction protein Lnk | | |  | | | | | |  | | 2.51 |  |
| MAP3K8 | | mitogen-activated protein 3 kinase 8 | | |  | | | | | |  | | 2.3 |  |
| PPP1R15A | | GADD34 | | |  | | | | | |  | | 2.07 |  |
| CXXC5 | | Putative MAPK-activating protein PM08 | | |  | | | | | |  | | -2.04 |  |
| ITPKA | | inositol 1,4,5-trisphosphate 3-kinase A | | |  | | | | | |  | | -2.04 |  |
|  | |  | | |  | | | | | |  | |  |  |
| **Transcription factors or modulators** | | | | |  | | | | | |  | |  | |
| CEBPD | | CCAAT/enhancer binding protein, delta | | |  | | | | | |  | | 7.18 |  |
| HOXD1 | | HOX4G | | |  | | | | | |  | | 4.29 |  |
| EDN1 | | endothelin 1 | | |  | | | | | |  | | 4.04 |  |
| NFKB1 | | NF-kappaB | | |  | | | | | |  | | 3.79 |  |
| NCOA7 | | nuclear receptor coactivator 7 | | |  | | | | | |  | | 2.99 |  |
| RELB | | v-rel reticuloendotheliosis viral oncogene homolog B | | | | | | | | |  | | 2.97 |  |
| NFKBIA | | IkBa | | | | | | |  | |  | | 2.92 |  |
| EGR1 | | early growth response 1 | | | | | | |  | |  | | 2.8 |  |
| RASD1 | | dexamethasone-induced Ras-related protein | | | | | | |  | |  | | 2.8 |  |
| ELF3 | | ERT | | | | | | |  | |  | | 2.69 |  |
| ZC3H12A | | MCP induced protein 1 | | | | | | |  | |  | | 2.55 |  |
| ID1 | | inhibitor of DNA binding 1 | | | | | | |  | |  | | 2.49 |  |
| CEBPB | | CCAAT/enhancer binding protein, beta | | | | | | |  | |  | | 2.37 |  |
| SMAD3 | | Mothers against DPP homolog 3 | | | | | | |  | |  | | 2.31 |  |
| IKBKE | | IKK-i | | | | | | |  | |  | | 2.28 |  |
| ID3 | | inhibitor of DNA binding 3 | | | | | | |  | |  | | 2.17 |  |
| JUNB | | Jun-B oncogene | | | | | | |  | |  | | 2.13 |  |
| ZFPM1 | | FOG1 | | | | | | |  | |  | | -2.08 |  |
| MYCN | | v-myc myelocytomatosis viral related oncogene | | | | | | | | |  | | -2.78 |  |
| CEBPA | | CCAAT/enhancer binding protein, alpha | | | |  | | | | |  | | -2.86 |  |
|  | |  | | | |  | | | | |  | |  |  |
| **Transporter** | |  | | | |  | | | | |  | |  |  |
| SLC2A6 | | solute carrier family 2, member 6 | | | |  | | | | |  | | 6.32 |  |
| SLC6A14 | | solute carrier family 6, member 14 | | | |  | | | | |  | | 2.27 |  |
| SLC7A1 | | solute carrier family 7, member 1 | | | |  | | | | |  | | 2.06 |  |
| SLC20A1 | | solute carrier family 20, member 1 | | | |  | | | | |  | | 2.04 |  |
|  |  | | | | | | |  | | | |  |  |  |
| **Gene name** | **Alias or gene description** | | | | | | |  | | | |  | **GENK treatment**  **(fold change)** |  |
|  |  | | | | | | |  | | | |  |  |  |
| **Others** |  | | | | | | |  | | | |  |  |  |
| TNFAIP2 | TNF alpha-induced protein 2 | | | | | | |  | | | |  | 4.39 |  |
| SOD2 | superoxide dismutase 2, mitochondrial | | | | | | |  | | | |  | 4.1 |  |
| RND1 | Rho family GTPase 1 | | | | | | |  | | | |  | 3.37 |  |
| SERPINA3 | serpin peptidase inhibitor, clade A | | | | | | |  | | | |  | 3.35 |  |
| TRIM15 | tripartite motif containing 15 | | | | | | |  | | | |  | 2.87 |  |
| CFB | complement factor B | | | | | | |  | | | |  | 2.85 |  |
| GCNT3 | glucosaminyl (N-acetyl) transferase 3, mucin type | | | | | | | | | | |  | 2.62 |  |
| NAV3 | neuron navigator 3 | | | | | | | | |  | |  | 2.6 |  |
| SESTD1 | SEC14 and spectrin domains 1 | | | | | | | | |  | |  | 2.45 |  |
| TNIP1 | TNFAIP3 interacting protein 1 | | | | | | | | |  | |  | 2.39 |  |
| PIK3AP1 | phosphoinositide-3-kinase adaptor protein 1 | | | | | | | | |  | |  | 2.29 |  |
| TBC1D9 | TBC1 domain family, member 9 | | | | | | | | |  | |  | 2.14 |  |
| GBP2 | guanylate binding protein 2, interferon-inducible | | | | | | | | | | |  | 2.12 |  |
| CD38 | Cyclic ADP-ribose hydrolase 1 | | |  | | | | | | | |  | 2.11 |  |
| FAM107B | family with sequence similarity 107, member B | | | | | | | | | | |  | 2.1 |  |
| SAA4 | serum amyloid A4 | | |  | | | | | | | |  | 2.1 |  |
| FAM43A | family with sequence similarity 43, member A | | | | | | | | | | |  | 2.08 |  |
| ACSL1 | acyl-CoA synthetase long-chain family member 1 | | | | | | | | | | |  | 2.07 |  |
| ERRFI1 | ERBB receptor feedback inhibitor 1 | | | | | | |  | | | |  | 2.06 |  |
| GADD45A | growth arrest and DNA-damage-inducible, alpha | | | | | | | | | | |  | 2.04 |  |
| NAMPT | nicotinamide phosphoribosyltransferase | | | | | | | | |  | |  | 2.04 |  |
| SLC25A42 | solute carrier family 25, member 42 | | | | | | | | |  | |  | -2 |  |
| NAT8B | N-acetyltransferase 8B | | | | | | | | |  | |  | -2.08 |  |
| PLCXD1 | phosphatidylinositol-specific phospholipase C | | | | | | | | | | |  | -2.13 |  |

|  |  |  |  |
| --- | --- | --- | --- |
