# Supplementary material for: A Daphnane Diterpenoid Isolated from Wikstroemia polyantha Induces an Inflammatory Response and Modulates miRNA Activity
Source: PLoS One. 2012 Jun 26;7(6):e39621. doi: 10.1371/journal.pone.0039621 (PMC3383676; doi:10.1371/journal.pone.0039621)
Supplement: Table S3 — List of genes up-regulated at both 1 and 4 hours treatment with GENK (fold change threshold of 2, adjusted p-value of 0.01). (DOCX) [file pone.0039621.s007.docx]

**Table S3**

| **Gene name** | **Alias or gene description** | **1 hr** | **4 hrs** |
| --- | --- | --- | --- |
|  |  |  |  |
| **Cell death, survival** |  |  |  |
| C8ORF4 | chromosome 8 open reading frame 4 | 3.34 | 6.96 |
| PHLDA1 | pleckstrin homology-like domain | 3.03 | 5.63 |
|  |  |  |  |
| **Chemokine or cytokine** |  |  |  |
| CXCL2 | GRO2 | 13.37 | 5.26 |
| IL8 | interleukin 8 | 9.68 | 17.54 |
| CXCL1 | Gro1 | 5.49 | 7.75 |
| CCL20 | MIP3A | 3.18 | 5.07 |
| CXCL10 | IP-10 | 2.01 | 10.75 |
|  |  |  |  |
| **Signal Transduction** |  |  |  |
| TNFAIP3 | A20 | 7.7 | 6.74 |
| IER3 | immediate early response 3 | 7.52 | 4.39 |
| SGK | serum/glucocorticoid regulated kinase 1 | 3.25 | 2.79 |
| PPP1R15A | GADD34 | 3.09 | 2.07 |
| MAP3K8 | mitogen-activated protein 3 kinase 8 | 2.01 | 2.3 |
|  |  |  |  |
| **Transcription factors or modulators** | |  |  |
| EGR1 | early growth response 1 | 12.95 | 2.8 |
| RASD1 | dexamethasone-induced Ras-related protein | 7.44 | 2.8 |
| NFKBIA | IkBa | 4.56 | 2.92 |
| JUNB | Jun-B oncogene | 2.41 | 2.13 |
| ELF3 | Epithelial-restricted with serine box | 2.01 | 2.69 |
|  |  |  |  |
| **Others** |  |  |  |
| EDN1 | endothelin 1 | 2.21 | 4.04 |
