# Supplementary material for: A Daphnane Diterpenoid Isolated from Wikstroemia polyantha Induces an Inflammatory Response and Modulates miRNA Activity
Source: PLoS One. 2012 Jun 26;7(6):e39621. doi: 10.1371/journal.pone.0039621 (PMC3383676; doi:10.1371/journal.pone.0039621)
Supplement: Materials S1 — (DOC) [file pone.0039621.s008.doc]

**Supplemental Material SI**

Page SI-2: General experimental procedures

Page SI-3: Isolation procedure

Page SI-4: Derivatization of genkwanine M (**1**) to diacetyl genkwanine M (**2**)

Page SI-5: Table 1. NMR data for genkwanine M (**1**) recorded in DMSO-*d_6_* at 600 MHz

Page SI-6: Table 2. NMR data for diacetyl genkwanine M (**2**) recorded in DMSO-*d_6_* at 600 MHz

Page SI-7: Table 3. NMR data for genkwanine P (**3**) recorded in DMSO-*d_6_* at 600 MHz

Page SI-8: Structures of genkwanine M (**1**), diacetyl genkwanine M (**2**), genkwanine P (**3**)

Page SI-9: ^1^H spectrum of genkwanine M (**1**) recorded in DMSO-*d_6_* at 600 MHz

Page SI-10: ^13^C spectrum of genkwanine M (**1**) recorded in DMSO-*d_6_* at 150 MHz

Page SI-11: ^1^H spectrum of diacetyl genkwanine M (**2**) recorded in DMSO-*d_6_* at 600 MHz

Page SI-12: ^13^C spectrum of diacetyl genkwanine M (**2**) recorded in DMSO-*d_6_* at 150 MHz

Page SI-13: ^1^H spectrum of genkwanine P (**3**) recorded in DMSO-*d_6_* at 600 MHz

Page SI-14: ^13^C spectrum of genkwanine P (**3**) recorded in DMSO-*d_6_* at 150 MHz

Page SI-15: ROESY correlations of genkwanine M (**1**)

Page SI-16: ROESY spectrum of genkwanine M (**1**) recorded in DMSO-*d_6_* at 600 MHz

Page SI-17: ORTEP diagram of diacetyl genkwanine M (**2**)

Page SI-18/23: X-ray structure report

Page SI-24: NMR assignment of genkwanine M (**1**)

Page SI-25: NMR assignment of diacetyl genkwanine M (**2**)

Page SI-26: NMR assignment of genkwanine P (**3**)

SI-2

**General Experimental Procedures.** Optical rotations were measured using a Jasco P-1010 spectrophotometer. The ^1^H and ^13^C NMR spectra were recorded on a Bruker AV-600 spectrometer with a 5 mm CPTCI cryoprobe. ^1^H chemical shifts are referenced to the residual DMSO- *d_6_* signal (δ 2.50 ppm) and ^13^C chemical shifts are referenced to the DMSO- *d_6_* solvent peak (δ 39.51 ppm). The following abbreviations were used to explain the multiplicities: s = singlet, d = doublet, t = triplet, q = quartet, m = multiplet, quin = quintuplet, sext = sextet, sep = septet, b = broad. Low resolution ESI +/- were recorded on Bruker Esquire LC ion trap mass spectrometer equipped with an electrospray ion source. The solvent for ESI-MS experiments was methanol. The sample solution concentration was 10μM. It was infused into the ion source by a syringe pump at flow rate of 10 μL/min. High resolution ESI+ were recorded on a Micromass LCT time-of-flight (TOF) mass spectrometer equipped with an electrospray ion source. The samples were dissolved in MeOH. The working solutions were 20μM. Flow rate: 20μL min-1; sample cone: 90V; source temperature: 120 ºC; desolvation temperature: 120 ºC. The mass of arg-lys-asp-val-tyr was used as lock mass for genkwanine M (**1**), and the mass of erythromycin as lock mass for diacetyl genkwanine M (**2**). Merck Type 5554 silica gel plates were used for analytical thin layer chromatography. Sephadex® LH-20 column packed and elueted with a mixture of 1:1 MeOH/CH_2_Cl_2_ was used for size separation chromatography. Reversed-phase HPLC purifications were performed on a Waters 600E System Controller liquid chromatography attached to a Waters 996 photodiode array detector using a C_18_ reversed-phase column (CSC-Inertsil 150A/ODS2, 5 µm 25 x 0.94 cm). All solvents used for HPLC were Fisher HPLC grade. The acetylation reaction of the genkwanine M was carried out under a nitrogen atmosphere with dry solvents under anhydrous conditions. Commercially available anhydrous tetrahydrofuran (THF) was used to perform the reaction. Yield refer to chromatographically and spectroscopically (^1^H NMR) homogeneous materials. Reagents were purchased at the highest commercial quality and used without further purification. The reaction was monitored by thin layer chromatography (TLC) carried out on Merck Type 5554 silica gel plates using UV light as visualizing agent and either an ethanolic solution of cerium sulfate or vanillin in ethanol/aqueous H_2_SO_4_, and heat as developing agents.

SI-3

**Isolation Procedure.** Leaves and twigs from a 0.5 m tall shrub of *Wikstroemia polyantha* (family Thymelaeaceae) were collected on the centerline of Peninsula Malaysia approximately 50 miles south of the Thai border by E. Soepadmo and M. Suhaimi under contract with the University of Chicago at Illinois. A voucher numbered Q66O4184 was deposit in the Field Museum in Chicago and at the National Herbarium at the Smithsonian. The plant material was transferred to the NCI Open Repository where it was dried and then extracted with CH_2_Cl_2_ and MeOH. The crude extract was entered into the Open Repository screening plates as sample number NO44759. Three grams of the crude extract were supplied to UBC by D. Newman of the NCI Open Repository. Three grams of the crude extract were then partitioned between EtOAc (3×50mL) and H_2_O (100 mL). The combined EtOAc extract was evaporated to dryness to give 2.1 g of dark green oil; 1.000 g of this was chromatographed on a Waters 10g normal phase Sep-Pak employing a step gradient from 95:5 *n*-Hexanes/EtOAc to EtOAc, and from 90:10 EtOAc/MeOH to MeOH, to give fractions A-E. Fraction C (38 mg), eluting with 60:40 *n*-Hexanes/EtOAc, was chromatographed on a Sephadex® LH-20 column using 1:1 MeOH/CH_2_Cl_2_ as an eluent to give fractions C-(A-G). Pure genkwanine M (**1**) (4.9 mg) and genkwanine P (**3**) (2.1 mg) were obtained as a white amorphous solids from fraction C-E (15 mg) via C_18_ reversed-phase HPLC using 8:2 MeOH/H_2_O as an eluent over 70 min. (flow rate 2 mL/min).

SI-4

**Derivatization of genkwanine M (1).** To a stirred solution of acetic anhydride (4µL, 4.2x10^-5^ mol) and DMAP (5.2x10^-5^g, 4.2x10^-7^ mol) in anhydrous THF (0.5 mL) was added genkwanine M (**1**) (2.5 x10^-3^g, 4.2x10^-6^ mol), previously dissolved in anhydrous THF (0.5 mL), and the mixture was stirred for 24 h at rt. Then the mixture of acetylated products was concentrated to dryness and purified by C_18_ reversed-phase HPLC using a CSC-Inertsil 150A/ODS2, 5 μm 25 x 0.94 cm column and 80:20 MeOH/H_2_O as an eluent to yield pure diacetyl genkwanine M as major product (1.1mg). NMR data obtained for the diacetyl product was consistent with acetylation at OH-3 and OH-5 of genkwanine M (**1**). Diacetyl genkwanine M (**2**) gave needle-shaped crystals from a mixture of 9:1 CCl_4_/Hexane.

SI-5

**Table 1.** NMR Spectroscopic Data (600MHz, DMSO-*d6*) for genkwanine M (**1**)

|  | | | |
| --- | --- | --- | --- |
| genkwanine M (**1**) | | | |
| position | δ_C_, multi. | δ_H_, (*J* in Hz) | HMBC*^a^* |
|  | | | |
| 1 | 34.5, CH_2_ | 1.49, m | 2, 3, 9, 10 |
| 2 | 36.1, CH | 1.52, m | 1, 4, 10, 19 |
| 3 | 76.4, CH | 3.68, t (5.4) | 1, 5, 10 |
| 4 | 79.5, C |  |  |
| 5 | 71.2, CH | 3.73, d (9.6) | 4, 6, 7 |
| 6 | 60.0, C |  |  |
| 7 | 63.4, CH | 3.49, s | 6, 8, 9, 14, 20 |
| 8 | 35.9, CH | 3.04, d (3.0) | 6, 7, 11 |
| 9 | 80.1, C |  |  |
| 10 | 48.1, CH | 2.52, m*^b^* | 1, 4, 5, 11 |
| 11 | 34.4, CH | 2.41, quin (7.2) | 9, 10, 12, 13, 18 |
| 12 | 35.6, CH_2_ | 2.18, dd (14.4, 7.2) | 9, 11, 13, 18 |
|  |  | 1.58, d (14.4) |  |
| 13 | 84.0, C |  |  |
| 14 | 81.2, CH | 4.70, d (3.0) | 1’, 7, 9, 15 |
| 15 | 146.4, C |  |  |
| 16 | 110.5, CH_2_ | 5.03, bs | 13, 15, 17 |
|  |  | 4.87, bs |  |
| 17 | 19.0, CH_3_ | 1.79, s | 13, 15, 16 |
| 18 | 20.8, CH_3_ | 1.17, d (7.2) | 9, 11, 12 |
| 19 | 13.5, CH_3_ | 0.94, d (6.6) | 1, 2, 3 |
| 20 | 67.8, CH_2_ | 4.95, d (12.0) | 1”, 5, 6, 7 |
|  |  | 3.92, d (12.0) |  |
| 1’ | 116.3, C |  |  |
| 2’ | 136.6, C |  |  |
| 3’ 7’ | 125.8, CH | 7.58, m | 1’, 5’ |
| 4’ 6’ | 127.8, CH | 7.38, m | 2’, 3’, 7’ |
| 5’ | 129.1, CH | 7.38, m | 2’, 3’, 7’ |
| 1” | 165.5, C |  |  |
| 2” | 129.8, C |  |  |
| 3” 7” | 129.3, CH | 8.01, d (7.8) | 1”, 2”, 5” |
| 4” 6” | 128.7, CH | 7.54, t (7.8) | 2”, 3”, 7” |
| 5” | 133.3, CH | 7.66, t (7.8) | 3”, 7” |
| 3-OH |  | 5.56, (5.4) | 2, 3, 4 |
| 4-OH |  | 4.25, s | 3, 4, 5, 10 |
| 5-OH |  | 4.94, d (9.6) | 4, 5 ,6 |
| *^a^*HMBC correlations, optimized for 10 Hz, are from proton(s) stated to the indicated carbon. | | | |
| *^b^*Signal partially obscured. | | | |

SI-6

**Table 2.** NMR Spectroscopic Data (600MHz, DMSO-*d6*) for diacetyl genkwanine M (**2**)

|  | | | |
| --- | --- | --- | --- |
| diacetyl genkwanine M (**2**) | | | |
| position | δ_C_, multi. | δ_H_, (*J* in Hz) | HMBC^a^ |
|  |  |  |  |
| 1 | 35.3, CH_2_ | 1.79, m*^b^* | 2, 10 |
|  |  | 1.56, dd (12.6, 12.0) |  |
| 2 | 35.0, CH | 1.69, q (4.8) | 1, 19 |
| 3 | 77.7, CH | 4.77, d (4.8) | 2, 4, 5, 10, 23 |
| 4 | 80.9, C |  |  |
| 5 | 71.4, CH | 5.33, s | 3, 6, 7, 21 |
| 6 | 58.7, C |  |  |
| 7 | 64.0, CH | 3.61, s | 6, 8, 9, 14, 20 |
| 8 | 36.0, CH | 3.16, m | 6, 7, 9, 11, 14 |
| 9 | 79.6, C |  |  |
| 10 | 48.4, CH | 2.62, m | 1, 2, 4, 5, 9, 11 |
| 11 | 34.4, CH | 2.48, m*^b^* | 9, 12, 13, 18 |
| 12 | 35.6, CH_2_ | 2.19, dd (14.1, 8.4) | 9, 11, 13, 14, 15 |
|  |  | 1.64, d (14.1) |  |
| 13 | 84.1, C |  |  |
| 14 | 81.0, CH | 4.75, d (2.4) | 7, 9, 15 |
| 15 | 146.2, C |  |  |
| 16 | 110.7, CH_2_ | 5.04, bs | 13, 15, 17 |
|  |  | 4.90, bs |  |
| 17 | 18.9, CH_3_ | 1.80, s | 13, 15, 16 |
| 18 | 20.7, CH_3_ | 1.21, d (6.6) | 11, 12 |
| 19 | 12.9, CH_3_ | 0.83, d (4.8) | 1, 2, 3 |
| 20 | 68.1, CH_2_ | 4.68, d (12.0) | 1”, 5, 6 |
|  |  | 3.93, d (12.0) |  |
| 21 | 170.0, C |  |  |
| 22 | 20.8, CH_3_*^c^* | 2.00, s | 21 |
| 23 | 170.1, C |  |  |
| 24 | 20.9, CH_3_*^c^* | 2.05, s | 23 |
| 1’ | 116.4, C |  |  |
| 2’ | 136.3, C |  |  |
| 3’ 7’ | 125.7, CH | 7.59, m | 1’, 5’ |
| 4’ 6’ | 127.9, CH | 7.39, m | 2’, 3’, 7’ |
| 5’ | 129.2, CH | 7.39, m | 2’, 3’, 7’ |
| 1” | 165.4, C |  |  |
| 2” | 129.2, C |  |  |
| 3” 7” | 129.4, CH | 7.98, d (7.2) | 1”, 2”, 5” |
| 4” 6” | 128.7, CH | 7.54, t (7.2) | 2”, 3”, 7” |
| 5” | 133.5, CH | 7.67, t (7.2) | 3”, 7” |
| 4-OH |  | 4.68, s | 3, 4, 5 |
| *^a^*HMBC correlations, optimized for 10 Hz, are from proton(s) stated to the indicated carbon. | | | |
| *^b^*Signal partially obscured. | | | |
| *^c^*May be interchanged. |  |  |  |

SI-7

**Table 3.** NMR Spectroscopic Data (600MHz, DMSO-*d6*) for genkwanine P (**3**)

|  | | | |
| --- | --- | --- | --- |
| genkwanine P (**3**) | | | |
| position | δ_C_, multi. | δ_H_, (*J* in Hz) | HMBC*^a^* |
|  | | | |
| 1 | 34.5, CH_2_ | 1.49, m | 2, 3, 9, 10 |
| 2 | 36.2, CH | 1.52, m | 1, 4, 19 |
| 3 | 76.4, CH | 3.67, m | 1, 5, 10 |
| 4 | 79.4, C |  |  |
| 5 | 71.2, CH | 3.66, m | 4, 6, 7 |
| 6 | 59.9, C |  |  |
| 7 | 63.3, CH | 3.45, s | 6, 8, 9, 20 |
| 8 | 35.9, CH | 3.02, d (2.4) | 7, 11 |
| 9 | 80.1, C |  |  |
| 10 | 48.1, CH | 2.53, m*^b^* | 1, 4, 5, 11 |
| 11 | 34.4, CH | 2.41, quin (7.8) | 9, 12, 13, 18 |
| 12 | 35.6, CH_2_ | 2.18, dd (13.8, 7.8) | 9, 11, 13, 18 |
|  |  | 1.58, d (13.8) |  |
| 13 | 84.0, C |  |  |
| 14 | 81.2, CH | 4.69, d (2.4) | 1’, 7, 9, 15 |
| 15 | 146.4, C |  |  |
| 16 | 110.5, CH_2_ | 5.03, bs | 13, 15, 17 |
|  |  | 4.87, bs |  |
| 17 | 19.0, CH_3_ | 1.78, s | 13, 15, 16 |
| 18 | 20.7, CH_3_ | 1.17, d (7.2) | 9, 11, 12 |
| 19 | 13.5, CH_3_ | 0.94, d (6.6) | 1, 2, 3 |
| 20 | 67.1, CH_2_ | 4.79, d (11.4) | 1”, 5, 6, 7 |
|  |  | 3.85, d (11.4) |  |
| 1’ | 116.3, C |  |  |
| 2’ | 136.6, C |  |  |
| 3’ 7’ | 125.8, CH | 7.58, m | 1’, 5’ |
| 4’ 6’ | 127.8, CH | 7.40, m | 2’, 3’, 7’ |
| 5’ | 129.1, CH | 7.40, m | 2’, 3’, 7’ |
| 1” | 166.1, C |  |  |
| 2” | 118.1, CH | 6.67, d (16.2) | 1”, 3”, 4” |
| 3” | 144.6, CH | 7.67, d (16.2) | 1”, 4” |
| 4” | 134.0, C |  |  |
| 5” 9” | 128.4, CH | 7.74, m | 3”, 7” |
| 6” 8” | 128.9, CH | 7.43, m | 4”, 5”, 9” |
| 7” | 130.5, CH | 7.43, m | 4”, 5”, 9” |
| 3-OH |  | 5.58, (5.4) | 2, 3, 4 |
| 4-OH |  | 4.24, s | 3, 4, 5, 10 |
| 5-OH |  | 4.87, d (9.6) | 4, 5 ,6 |
| *^a^*HMBC correlations, optimized for 10 Hz, are from proton(s) stated to the indicated carbon. | | | |
| *^b^*Signal partially obscured. | | | |

SI-8

Structure of genkwanine M (**1**)

Structure of diacetyl genkwanine M (**2**)

Structure of genkwanine P (**3**)

SI-9

^1^H NMR spectrum of genkwanine M (**1**) recorded in DMSO-*d_6_* at 600 MHz

SI-10

^13^C NMR spectrum of genkwanine M (**1**) recorded in DMSO-*d_6_* at 150 MHz

SI-11

^1^H NMR spectrum of diacetyl genkwanine M (**2**) recorded in DMSO-*d_6_* at 600 MHz

SI-12

^13^C NMR spectrum of diacetyl genkwanine M (**2**) recorded in DMSO-*d_6_* at 150 MHz

SI-13

^1^H NMR spectrum of genkwanine P (**3**) recorded in DMSO-*d_6_* at 600 MHz

SI-14

^13^C NMR spectrum of genkwanine P (**3**) recorded in DMSO-*d_6_* at 150 MHz

SI-15

ROESY correlations for genkwanine M (**1**). Genkwanine M (**1**) 3D structure by ChemBio3D Ultra®

SI-16

ROESY spectrum of genkwanine M (**1**) recorded in DMSO-*d_6_* at 600 MHz

SI-17


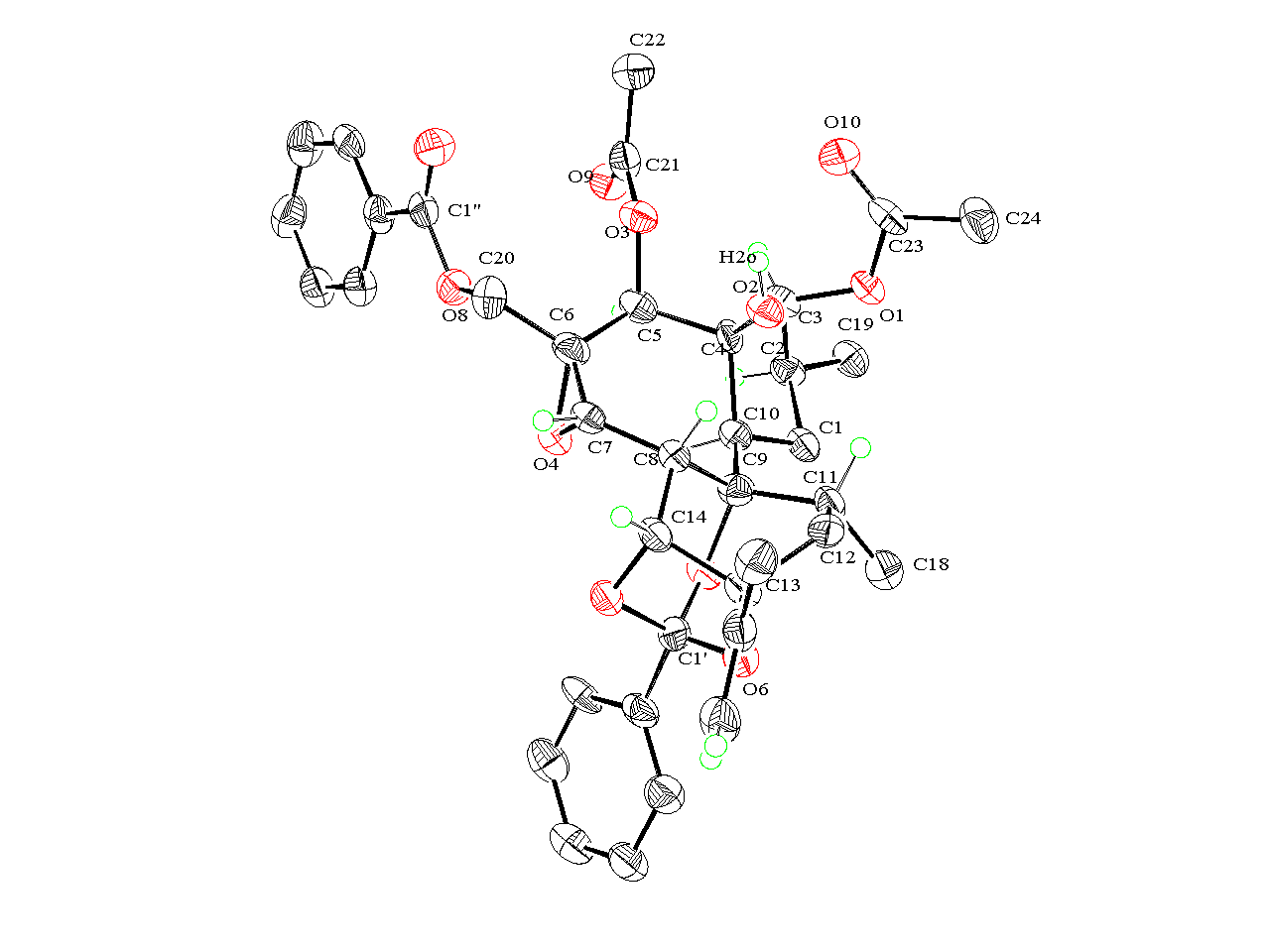


ORTEP diagram for diacetyl genkwanine M (**2**)

SI-18

## Experimental

# Data Collection

A colourless needle crystal of C_38_H_42_O_11_ having approximate dimensions of 0.02 x 0.05 x 0.40 mm was mounted on a glass fiber. All measurements were made on a Bruker DUO APEX II diffractometer with graphite monochromated Cu-Kα radiation.

The data were collected at a temperature of -183.0 + 0.1oC to a maximum 2θ value of 112.1o. Data were collected in a series of φ and ω scans in 1.00o oscillations with 120-second exposures. The crystal-to-detector distance was 50.00 mm.

Data Reduction

Of the 13842 reflections that were collected, 4344 were unique (Rint = 0.136); equivalent reflections were merged. Data were collected and integrated using the Bruker SAINT^1^ software package. The linear absorption coefficient, μ, for Mo-Kα radiation is 8.03 cm^-1^. Data were corrected for absorption effects using the multi-scan technique (SADABS^2^), with minimum and maximum transmission coefficients of 0.731 and 0.984, respectively. The data were corrected for Lorentz and polarization effects.

# Structure Solution and Refinement

The structure was solved by direct methods^3^. All non-hydrogen atoms were refined anisotropically. Hydrogen H2O was located in a difference map and refined isotropically. All other hydrogen atoms were placed in calculated positions. The absolute configuration was determined on the basis of the refined Flack parameter. 12 The final cycle of full-matrix least-squares refinement^4^ on F^2^ was based on 4344 reflections and 452 variable parameters and converged (largest parameter shift was 0.00 times its esd) with unweighted and weighted agreement factors of:

R1 = Σ ||Fo| - |Fc|| / Σ |Fo| = 0.112

wR2 = [ Σ ( w (Fo^2^ - Fc^2^)^2^ )/ Σ w(Fo^2^)^2^]1/2 = 0.140

SI-19

The standard deviation of an observation of unit weight^5^ was 1.00. The weighting scheme was based on counting statistics. The maximum and minimum peaks on the final difference Fourier map corresponded to 0.24 and –0.21 e‑/Å^3^, respectively.

Neutral atom scattering factors were taken from Cromer and Waber^6^. Anomalous dispersion effects were included in Fcalc^7^; the values for Δf' and Δf" were those of Creagh and McAuley^8^. The values for the mass attenuation coefficients are those of Creagh and Hubbell^9^. All refinements were performed using the SHELXL-9710 via the WinGX^11^ interface.

*References*

(1) SAINT. Version 7.68A. Bruker AXS Inc., Madison, Wisconsin, USA. (1997-2010).

(2) SADABS. Bruker Nonius area detector scaling and absorption correction - V2008/1, Bruker AXS Inc., Madison, Wisconsin, USA (2008).

(3) SIR97 - Altomare A., Burla M.C., Camalli M., Cascarano G.L., Giacovazzo C. , Guagliardi A., Moliterni A.G.G., Polidori G.,Spagna R. (1999) J. Appl. Cryst. 32, 115-119.

(4) Least Squares function minimized:

Σ*w*(Fo^2^-Fc^2^)^2^

(5) Standard deviation of an observation of unit weight:

[Σ*w*(Fo^2^-Fc^2^)2/(No-Nv)]1/2

where: No = number of observations

Nv = number of variables

(6) Cromer, D. T. & Waber, J. T.; "International Tables for X-ray Crystallography", Vol. IV, The Kynoch Press, Birmingham, England, Table 2.2 A (1974).

(7) Ibers, J. A. & Hamilton, W. C.; Acta Crystallogr., 17, 781 (1964).

(8) Creagh, D. C. & McAuley, W.J .; "International Tables for Crystallography", Vol C, (A.J.C. Wilson, ed.), Kluwer Academic Publishers, Boston, Table 4.2.6.8, pages 219-222 (1992).

(9) Creagh, D. C. & Hubbell, J.H..; "International Tables for Crystallography", Vol C, (A.J.C. Wilson, ed.), Kluwer Academic Publishers, Boston, Table 4.2.4.3, pages 200-206 (1992).

SI-20

(10) SHELXTL Version 5.1. Bruker AXS Inc., Madison, Wisconsin, USA. (1997).

(11) WinGX – V1.70 – Farrugia, L.J.; J. Appl. Cryst., 32, 837 (1999).

(12) FLACK X-PARAMETER - (a) Flack, H.D. Acta Crystallogr., Sect A 1983, 39, 876-881. (b) Bernardinelli, G.; Flack, H. D. Acta Crystallogr., Sect A 1985, 41, 500-511.

SI-21

*EXPERIMENTAL DETAILS*

A. Crystal Data

Empirical Formula C_38_H_42_O_11_

Formula Weight 674.72

Crystal Colour, Habit colourless, needle

Crystal Dimensions 0.02 X 0.05 X 0.40 mm

Crystal System orthorhombic

Lattice Type primitive

Lattice Parameters a = 12.072(1) Å

b = 15.673(1) Å

c = 17.874(1) Å

α = 90 o

β = 90 o

γ = 90 o

V = 3381.6(5) Å^3^

Space Group *P* 2_1_2_1_2_1_ (#19)

Z value 4

Dcalc 1.325 g/cm^3^

F_000_ 1432.00

μ(MoKα) 8.05 cm^-1^

SI-22

B. Intensity Measurements

Diffractometer Bruker DUO APEX II

Radiation MoKα (λ = 0.71073 Å)

graphite monochromated

Data Images 1407 exposures @ 120.0 seconds

Detector Position 50.00 mm

2θmax 112.1o

No. of Reflections Measured Total: 13842

Unique: 4344(Rint = 0.136)

Corrections Absorption (T_min_ = 0.731, T_max_= 0.984)

Lorentz-polarization

SI-23

C. Structure Solution and Refinement

Structure Solution Direct Methods (SIR97)

Refinement Full-matrix least-squares on F^2^

Function Minimized Σ w (Fo^2^ - Fc^2^)^2^

Least Squares Weights w=1/(σ2(Fo^2^)+(0.0503P) 2+ 0.0000P)

Anomalous Dispersion All non-hydrogen atoms

No. Observations (I>0.00σ(I)) 4344

No. Variables 452

Reflection/Parameter Ratio 9.61

Residuals (refined on F^2^, all data): R1; wR2 0.112; 0.140

Goodness of Fit Indicator 1.00

No. Observations (I>2.00σ(I)) 2826

Residuals (refined on F): R1; wR2 0.058; 0.118

Max Shift/Error in Final Cycle 0.00

Maximum peak in Final Diff. Map 0.24 e‑/Å^3^

Minimum peak in Final Diff. Map -0.21 e‑/Å^3^

SI-24

Genkwanine M (**1**)

[α]^25^_D_ -6.0° (c 0.02, MeOH); ^1^H NMR (DMSO-*d_6_*, 600 MHz) *δ* 8.01 (2H, d, *J* = 7.8 Hz, H-3”, H-7”), 7.66 (1H, t, *J* = 7.8 Hz, H-5”), 7.59-7.57 (2H, m, H-3’, H-7’), 7.54 (2H, t, *J* = 7.8 Hz, H-4”, H-6”), 7.39-7.37 (3H, m, H-4’, H-5’, H-6’), 5.56 (1H, d, *J* = 5.4 Hz, 3-OH), 5.03 (1H, bs, H-16_a_), 4.95 (1H, d, *J* = 12.0 Hz, H-20_a_), 4.94 (1H, d, *J* = 9.6 Hz, 5-OH), 4.87 (1H, bs, H-16_b_), 4.70 (1H, d, *J* = 3.0 Hz, H-14), 4.25 (1H, s, 4-OH), 3.92 (1H, d, *J* = 12.0 Hz, H-20_b_), 3.73 (1H, d, *J* = 9.6 Hz, H-5), 3.68 (1H, t, *J* = 5.4 Hz, H-3), 3.49 (1H, s, H-7), 3.04 (1H, d, *J* = 3.0 Hz, H-8), 2.52 (1H, m, H-10), 2.41 (1H, quin, *J* = 7.2, H-11), 2.18 (1H, dd, *J* = 14.4, 7.2 Hz, H-12_a_), 1.79 (3H, s, H-17), 1.58 (1H, d, *J* = 14.4, H-12_b_), 1.52 (1H, m, H-2), 1.49 (2H, m, H-1), 1.17 (3H, d, *J* = 7.2 Hz, H-18), 0.94 (3H, d, *J* = 6.6 Hz, H-19); ^13^C NMR (DMSO-*d_6_*, 150 MHz) *δ* 165.5 (C, C-1”), 146.4 (C, C-15), 136.6 (C, C-2’), 133.3 (CH, C-5”), 129.8 (C, C-2”), 129.3 (2CH, C-3”, C-7”), 129.1 (CH, C-5’), 128.7 (2CH, C-4”, C-6”), 127.8 (2CH, C-4’, C-6’), 125.8 (2CH, C-3’, C-7’), 116.3 (C, C-1’), 110.5 (CH_2_, C-16), 84.0 (C, C-13), 81.2 (CH, C-14), 80.1 (C, C-9), 79.5 (C, C-4), 76.4 (CH, C-3), 71.2 (CH, C-5), 67.8 (CH_2_, C-20), 63.4 (CH, C-7), 60.0 (C, C-6), 48.1 (CH, C-10), 36.1 (CH, C-2), 35.9 (CH, C-8), 35.6 (CH_2_, C-12), 34.5 (CH_2_, C-1), 34.4 (CH, C-11), 20.8 (CH_3_, C-18), 19.0 (CH_3_, C-17), 13.5 (CH_3_, C-19); HRESIMS [M + Na]^+^ *m/z* 613.2421 (calcd for C_34_H_38_O_9_Na, 613.2414).

SI-25

Diacetyl genkwanine M (**2**)

^1^H NMR (DMSO-*d_6_*, 600 MHz) *δ* 7.98 (2H, d, *J* = 7.2 Hz, H-3”, H-7”), 7.67 (1H, t, *J* = 7.2 Hz, H-5”), 7.60-7.58 (2H, m, H-3’, H-7’), 7.54 (2H, t, *J* = 7.2 Hz, H-4”, H-6”), 7.40-7.39 (3H, m, H-4’, H-5’, H-6’), 5.33 (1H, s, H-5), 5.04 (1H, bs, H-16_a_), 4.90 (1H, bs, H-16_b_), 4.77 (1H, d, *J* = 4.8 Hz, H-3), 4.75 (1H, d, *J* = 2.4 Hz, H-14), 4.68 (1H, d, *J* = 12.0 Hz, H-20_a_), 4.68 (1H, s, 4-OH), 3.93 (1H, d, *J* = 12.0 Hz, H-20_b_), 3.61 (1H, s, H-7), 3.16 (1H, m, H-8), 2.62 (1H, m, H-10), 2.48 (1H, m, H-11), 2.19 (1H, dd, *J* = 14.1, 8.4 Hz, H-12_a_), 2.05 (3H, s, H-24), 2.00 (3H, s, H-22), 1.80 (3H, s, H-17), 1.79 (1H, m, H-1_a_), 1.69 (1H, q, *J* = 4.8 Hz, H-2), 1.64 (1H, d, *J* = 14.1, H-12_b_), 1.56 (1H, dd, *J* = 12.6, 12.0 Hz, H-1_b_), 1.21 (3H, d, *J* = 6.6 Hz, H-18), 0.83 (3H, d, *J* = 4.8 Hz, H-19); ^13^C NMR (DMSO-*d_6_*, 150 MHz) *δ* 170.1 (C, C-23), *δ* 170.0 (C, C-21), 165.4 (C, C-1”), 146.2 (C, C-15), 136.3 (C, C-2’), 133.5 (CH, C-5”), 129.4 (2CH, C-3”, C-7”), 129.2 (C, C-2”), 129.2 (CH, C-5’), 128.7 (2CH, C-4”, C-6”), 127.9 (2CH, C-4’, C-6’), 125.7 (2CH, C-3’, C-7’), 116.4 (C, C-1’), 110.7 (CH_2_, C-16), 84.1 (C, C-13), 81.0 (CH, C-14), 80.9 (C, C-4), 79.6 (C, C-9), 77.7 (CH, C-3), 71.4 (CH, C-5), 68.1 (CH_2_, C-20), 64.0 (CH, C-7), 58.7 (C, C-6), 48.4 (CH, C-10), 36.0 (CH, C-8), 35.6 (CH_2_, C-12), 35.3 (CH_2_, C-1), 35.0 (CH, C-2), 34.4 (CH, C-11), 20.9 (CH_3_, C-24), 20.8 (CH_3_, C-22); 20.7 (CH_3_, C-18), 18.9 (CH_3_, C-17), 12.9 (CH_3_, C-19); HRESIMS [M + Na]^+^ *m/z* 697.2620 (calcd for C_38_H_42_O_11_Na, 697.2625).

SI-26

Genkwanine P (**3**)

[α]^25^_D_ +4.7° (c 0.02, MeOH); ^1^H NMR (DMSO-*d_6_*, 600 MHz) *δ* 7.74-7.73 (2H, m, H-5”, H-9”), 7.67 (1H, d, *J* = 16.2 Hz, H-3”), 7.59-7.57 (2H, m, H-3’, H-7’), 7.43-7.42 (3H, m, H-6”, H-7”, H-8”), 7.40-7.39 (3H, m, H-4’, H-5’, H-6’), 6.67 (1H, d, *J* = 16.2 Hz, H-2”), 5.58 (1H, d, *J* = 5.4 Hz, 3-OH), 5.03 (1H, bs, H-16_a_), 4.87 (1H, d, *J* = 9.6 Hz, 5-OH), 4.87 (1H, bs, H-16_b_), 4.79 (1H, d, *J* = 11.4 Hz, H-20_a_), 4.69 (1H, d, *J* = 2.4 Hz, H-14), 4.24 (1H, s, 4-OH), 3.85 (1H, d, *J* = 11.4 Hz, H-20_b_), 3.67 (1H, m, H-3), 3.66 (1H, m, H-5), 3.45 (1H, s, H-7), 3.02 (1H, d, *J* = 2.4 Hz, H-8), 2.53 (1H, m, H-10), 2.41 (1H, quin, *J* = 7.8, H-11), 2.18 (1H, dd, *J* = 13.8, 7.8 Hz, H-12_a_), 1.78 (3H, s, H-17), 1.58 (1H, d, *J* = 13.8, H-12_b_), 1.52 (1H, m, H-2), 1.49 (2H, m, H-1), 1.17 (3H, d, *J* = 7.2 Hz, H-18), 0.94 (3H, d, *J* = 6.6 Hz, H-19); ^13^C NMR (DMSO-*d_6_*, 150 MHz) *δ* 166.1 (C, C-1”), 146.4 (C, C-15), 144.6 (CH, C-3”), 136.6 (C, C-2’), 134.0 (C, C-4”), 130.5 (CH, C-7”), 129.1 (CH, C-5’), 128.9 (2CH, C-6”, C-8”), 128.4 (2CH, C-5”, C-9”), 127.8 (2CH, C-4’, C-6’), 125.8 (2CH, C-3’, C-7’), 118.1 (CH, C-2”), 116.3 (C, C-1’), 110.5 (CH_2_, C-16), 84.0 (C, C-13), 81.2 (CH, C-14), 80.1 (C, C-9), 79.4 (C, C-4), 76.4 (CH, C-3), 71.2 (CH, C-5), 67.1 (CH_2_, C-20), 63.3 (CH, C-7), 59.9 (C, C-6), 48.1 (CH, C-10), 36.2 (CH, C-2), 35.9 (CH, C-8), 35.6 (CH_2_, C-12), 34.5 (CH_2_, C-1), 34.4 (CH, C-11), 20.7 (CH_3_, C-18), 19.0 (CH_3_, C-17), 13.5 (CH_3_, C-19); HRESIMS [M + Na]^+^ *m/z* 639.2554 (calcd for C_36_H_40_O_9_Na, 639.2570).
